# Beehive Products as Antibacterial Agents: A Review

**DOI:** 10.3390/antibiotics10060717

**Published:** 2021-06-15

**Authors:** Rita Abou Nader, Rawan Mackieh, Rim Wehbe, Dany El Obeid, Jean Marc Sabatier, Ziad Fajloun

**Affiliations:** 1 Faculty of Sciences 3, Department of Biology, Lebanese University, Campus Michel Slayman Ras Maska, Tripoli 1352, Lebanon; ritaabounaderr@hotmail.com (R.A.N.); rawan97mck@gmail.com (R.M.); 2 Biology Department, Faculty of Arts and Sciences, American University of Beirut, Beirut 1107 2020, Lebanon; rgw00@mail.aub.edu; 3 Faculty of Agriculture & Veterinary Sciences, Lebanese University, Dekwaneh, Beirut 2832, Lebanon; delobeid@gmail.com; 4 Faculté de Médecine Secteur Nord, 51, Université Aix-Marseille, Institut de Neuro-Physiopathologie, UMR 7051, Boulevard Pierre Dramard-CS80011, CEDEX 15, 13344 Marseille, France; 5 Laboratory of Applied Biotechnology (LBA3B), Azm Center for Research in Biotechnology and its Applications, EDST, Lebanese University, Tripoli 1300, Lebanon

**Keywords:** antibacterial activity, honeybee products, honey, bee venom, propolis, royal jelly, pollen

## Abstract

Honeybees are one of the most marvelous and economically beneficial insects. As pollinators, they play a vital role in every aspect of the ecosystem. Beehive products have been used for thousands of years in many cultures for the treatment of various diseases. Their healing properties have been documented in many religious texts like the Noble Quran and the Holy Bible. Honey, bee venom, propolis, pollen and royal jelly all demonstrated a richness in their bioactive compounds which make them effective against a variety of bacterial strains. Furthermore, many studies showed that honey and bee venom work as powerful antibacterial agents against a wide range of bacteria including life-threatening bacteria. Several reports documented the biological activities of honeybee products but none of them emphasized on the antibacterial activity of all beehive products. Therefore, this review aims to highlight the antibacterial activity of honey, bee venom, propolis, pollen and royal jelly, that are produced by honeybees.

## 1. Introduction

**Figure 1:**
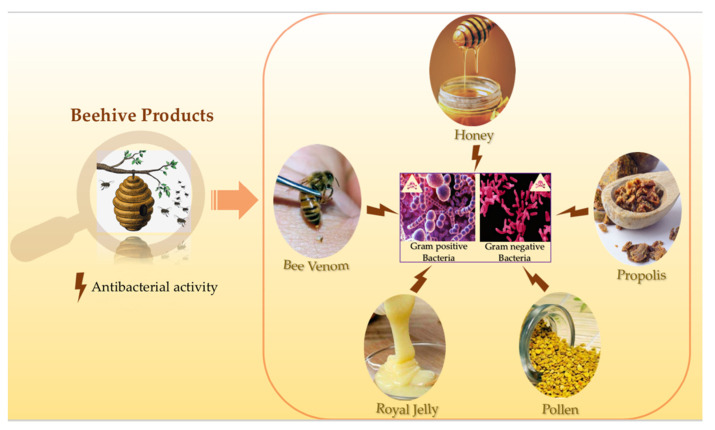
Schematic representation of the antibacterial properties of the beehive products.

Honeybees, also known as the ‘’Golden insect‘’, belong to the genus *Apis,* which is the Latin word for ‘’bee’’. These marvelous social insects live in a well-organized community and belong to the order Hymenoptera and to the family Apidae [1]. Honeybees can be found all around the world and are used for their vital role as pollinators in agriculture, but the main used species for crop pollination is *Apis mellifera* [2]. Since ancient times, honeybee products have been used for medicinal purposes. They have been also cited in many religious books [3]. Egyptians, Romans, Chinese and Persians have all documented for thousands of years the nutritional and medicinal values of bee products [4]. Apitherapy is a branch of unconventional medicine that relies on the usage of bee products which consist of honey, pollen, propolis, royal jelly and bee venom (BV). Besides having high nutritional importance and health benefits, honey showed antifungal, antiviral, antiseptic, anticancer, anti-diabetic, anti-inflammatory and cardio protective activities [5]. As for the BV, despite its possible adverse effects like the allergic reactions that might occur after the bee sting, one cannot disregard its various therapeutic effects. BV exerts anticancer, antiviral, antifungal and antibacterial effects, it is also used for the treatment of many neurodegenerative diseases [6,7,8,9,10]. Regarding propolis, it has the capacity to fight against cancer and many microorganisms [4,11,12]. Moreover, pollen possesses antioxidant, anticoagulant and anti-inflammatory properties [13,14,15]. Also, royal jelly exhibits several interesting biological activities including antioxidant [16], anti-aging [17], antitumor [18,19] anti-inflammatory, antimicrobial [20], and neurotrophic activities [21]. Hence, this review aims to highlight one of the most important and commonly shared biological activity of all of the above-mentioned beehive products, which is the antibacterial activity (Figure 1).

## 2. Antibacterial Activity of Bee Honey

Honey, one of the most well-known and highly valued natural products, is produced by honeybees (*Apis mellifera*) [22]. Since ancient times, honey has been used by mankind for medicinal and nutritional purposes [23]. 

Honey is a naturally occurring supersaturated sugar solution made up of a complex mixture of carbohydrates (77–86%), with fructose and glucose being the most abundant. It has a low amount of water (around 20%) which gives honey several of its attributes such as high viscosity and high osmotic pressure. It also contains numerous minor but essential components including proteins, enzymes (invertase, catalase, etc…), organic acids (gluconic acid, acetic acid), amino acids, lipids, vitamins, minerals and many others. Furthermore, honey from *Apis mellifera* is characterized by a low pH level ranging between 3.53 and 4.03 [22,23,24,25,26]. Nevertheless, honey composition varies widely depending on several factors like the floral origin, the environmental conditions, as well as the processing procedure it goes through (pasteurization and storage) [27,28,29].

The antibacterial effect of honey is mediated by two mechanisms: a peroxide-dependent and a peroxide-independent pathway. In the hydrogen peroxide (H_2_O_2_) pathway, H_2_O_2_ is formed by glucose oxidase, a carbohydrate metabolizing enzyme added to nectar by bees. As indicated by prior studies, the enzymatic oxidation of glucose is responsible for the antimicrobial activity of honey [30,31]. When samples of honey were treated with catalase, an enzyme that breaks down hydrogen peroxide, the bacterial growth was reduced [32,33]. Lastly, honey bacteriostatic DNA degradation was prevented when H_2_O_2_ was removed by catalase [34].

As for the peroxide independent pathway, the physicochemical properties consisting of high viscosity and high sugar content are the two factors that confer honey its antibacterial effect. In fact, the extraction of moisture from the surrounding environment causes bacterial dehydration by osmotic pressure. In a previous study of infants with gastroenteritis where glucose given in oral rehydration solution was substituted by honey, the recovery time of patients was significantly reduced [35]. This is likely due to the fact that the high sugar content in honey improves electrolyte and water reabsorption in the intestine. Moreover, the low pH level halts microbial growth [36,37]. Methylglyoxal (MGO) is another bioactive compound involved in non-peroxide antibacterial function. It is produced by non-enzymatic conversion of dihydroxyacetone (found in high concentration in the nectar of *Leptospermum scoparium flowers*) [38]. Manuka honey, a honey known to possess non-peroxide antibacterial activity due to MGO, is produced from the Myrtaceae family manuka tree, *L. scoparium* [39,40]. This honey has received a lot of attention from scientists around the world due to the biological properties it has, notably its antioxidant and antibacterial properties. Furthermore, Manuka honey has been used in the treatment of infections such as, surgical wounds, abscesses, traumatic wounds, burns, and ulcers of various origins and has demonstrated its potency in inhibiting human pathogens and prevent the formation of biofilms [41,42,43]. In addition to the bioactive compounds responsible of non-peroxide activity, an antimicrobial peptide present in honey called bee defensin-1 acts against several Gram-positive bacteria such as *Stephalococcus aureus, Bacillus subtilis*, and *Paenibacillus larvae*, the main cause of the ravaging American bee larvae disease [37]. 

With the emergence and spread of resistant pathogens, the properties of honey could be potentially clinically relevant. Honey demonstrates a wide range of antibacterial activity encompassing both Gram-negative and Gram-positive bacteria. In a previous study, *Escherichia coli* strains with ampicillin-resistance gene (β–lactamase) were found to be sensitive to honey [44]. In addition, Mokaya et al. collected 16 different types of honey from various Kenyan locations. They found that all of the 16 samples prevented *E. coli* growth and had a significant amount of non-peroxide antimicrobial activity [45]. Another study examined two natural honeys against Methicillin-resistant Staphylococcus aureus (MRSA), vancomycin-sensitive enterococci (VSE), and vancomycin-resistant enterococci (VRE) and then compared their antibacterial activity to that of artificial honey. All MRSA strains were resistant to artificial honey but sensitive to both honey types. As for VRE and VSE, both were susceptible to artificial and natural honey, with lower minimum inhibitory concentration (MIC) for natural honey [46]. These findings are in agreement with the study of Katrina et al. where MRSA and VRE were sensitive to the selected honeys and showed that the bacteriostatic effect was H_2_O_2_ dose-dependent [47]. 

In addition honey has been discovered to be a powerful inhibitor of *Helicobacter pylori,* causative agent of peptic ulcers [48]. 

As Manuka honey possesses an exceptional antibacterial activity, Brown et al. tried it against *Staphylococcus pseudintermedius* which cause serious infections in domesticated animals and can be transmitted to their owner. They demonstrated that Manuka honey is not just effective against novel multidrug-resistant *S. pseudintermedius* isolates, but additionally works in tandem with clinically important antibiotics and reduces its virulence [49]. 

A previous study has assessed the capacity of honey to prevent the adherence of *Salmonella interitidis* to the intestinal epithelial cells in vitro and has demonstrated that honey at dilutions 1:8 diminished the bacterial adherence from 25.6 ± 6.5 (control) to 6.7 ± 3.3 bacteria/epithelial cell [50].

Furthermore, another study found that a 10% concentration of honey could prevent oral bacteria like *Streptococcus mutans* from forming a biofilm, implying that honey could help to minimize oral pathogens in dental plaque [51]. Honey was also effective against biofilms produced by methicillin-susceptible *S. aureus* (MSSA), MRSA, and *Pseudomonas aeruginosa*, with bactericidal rates of 63–82%, 73–63%, and 91–91%, respectively, which were higher than the effect of widely used single antibiotics [52].

In another study, honey demonstrated antibacterial activity against *Burkholderia cepacia,* which is responsible of pulmonary infections especially in patients with cystic fibrosis and chronic granulomatous disease. Twenty strains of *B. cepacia* isolated from the sputum of patients with cystic fibrosis were screened for their susceptibility to eight antibiotics and two types of honey (with and without peroxide activity). All strains were resistant to the antibiotics tested but susceptible to honey at concentrations less than 6% (*v/v*) [53]. In addition, 13 honeys samples from different plant source were tested against *P. aeruginosa* and against *E. coli* at different concentrations (10%, 5%, 2.5%, and 1% *w/v*). All honey samples displayed an inhibitory effect on the growth of *P. aeruginosa* and *E. coli* for concentrations above 2.5%. Only four samples were still effective at a concentration of 2.5%: one against *E. coli* and three against *P. aeruginosa.* No activity was observed at concentrations below 2.5% [54]. 

The antimicrobial effect of honey has been tested against two Gram-positive organisms: *Streptococcus pyogenes* and *S. aureus*. Four honey samples were taken from beekeepers and tested at various concentrations (undiluted honey, 10 %, 30%, 50% and 70% *w/v*), then compared to standard antibiotics. Honey samples exhibited antibacterial activity with a diameter of the zone of inhibition (ZDI) ranging between 0 and 46 mm for *S. aureus* and between 0 and 44 mm for *S. pyogenes* [55]. Other studies also indicated the antibacterial activity of honey against human pathogens and foodborne pathogens such as *E.coli*, *Klebsiella pneumonia*, *Salmonella* spp., etc… [33,56].

Honey can be utilized to treat a wide range of oral diseases, including periodontal disease which is caused by *Porphyromonas gingivalis*, a Gram-negative bacterium. A previous study has shown that honey helps to prevent periodontal disease by killing anaerobic bacteria [57]. 

It is worth mentioning that the antibacterial activity of honey differs widely depending on the plant source [29,54]. Also, adulteration, thermal treatment, and long-term storage can all affect honey’s antibacterial function [58].

Fuertes et al. have explored the repressive mechanism of honey on the growth of bacteria. They found that when *E. coli* and *S. aureus* were exposed to different honey samples, physiological changes in membrane integrity and polarization occurred. Honey also caused a major metabolic disturbance in *S. aureus* as a primary physiological consequence [59]. 

Comparing the antibacterial potency of honey to that of antibiotics was also reported. A recent study compared the activity of three honeys with gentamicin against *E. coli* and *P. aeruginosa*. None diluted honey and its 1:2 to 1:6 aqueous dilutions had 100% and 96.4% activity against *P. aeruginosa* and *E. coli,* respectively. Gentamicin, on the other hand, showed lower antibacterial activity when used at concentrations of 8.0 and 4.0 g/mL [60]. 

Combining honey with other antibacterial compounds increases their inhibitory rate against microorganisms. In fact, when honey was combined with the gentamicin, the killing rate increased to more than 92–93% while the killing rate was around 77% for gentamicin alone and around 45% for honey alone [61]. Another study attempted to combine honey with ethanolic extract of cinnamon bark and found that the mixture had additive activity against acne-causing bacteria [62]. More studies have shown that the combination of honey with other bioactive compounds has increased its antibacterial activity [41,63,64,65].

Concerning clinical trials on honey, Blaser et al. were reported a full recovery in seven consecutive patients with MRSA infected or colonized wounds. Alternatively, antiseptics and antibiotics had previously failed to eradicate infection-related symptoms [66]. Furthermore, another study has demonstrated that consuming honey at least once a week considerably reduced the chance of *H. pylori* infection in a group of 150 dyspeptic patients [67]. Also, in a clinical trial involving 90 patients with infected wounds, non- gamma irradiated honey was associated with gradual decrease of bacterial load over a period of 4 weeks [68].

Studies used different methods to measure the antibacterial activity of honey. The most common methods were agar diffusion assay and serial dilution approach in microtiter plates. The first method, while being effortless and quick in performance, presents many limitations: (1) difficulty in loading a specific volume of the product sample into the agar wells due to the high viscosity of honey, (2) diffusion issues with active components such as defensin-1 and glucose oxidase, which have a large molecular weight across the agar matrix, (3) low reproducibility, (4) difficulty in comparing the results with those of other authors, (5) inability to discriminate between bacteriostatic and bactericidal activity. For the second method, serial dilution assay, the bacteriostatic (MIC) and bactericidal activity (MBC) of examined honey samples can be determined quantitatively using this approach in contrary to that of the first method. The only challenge that this method presents is the preparation of honey output solution [69].

In light of these findings, honey appears to exert wide antibacterial activity against a variety of microorganisms. It has been suggested in the literature that these properties make the development of honey-resistance unlikely and extrapolate that honey may have an important clinical utility in the treatment of antibiotic-resistant bacteria [70,71,72]. Also, the use of honey as an antibacterial agent has no negative side effects for patients, in contrast to antibiotics, and is cost effective [69]. Finally, honey may present some limitations when used as a treatment, like the presence of some toxic substances such as herbicides or pesticides in it, as well as its possible contamination by clostridium endospores. Therefore, it is crucial to follow specific criteria and standards when using honey as a therapeutic agent [23,73]. It is also mandatory to create a sterilizing procedure that is safe for honey proteinaceous antibacterial components (glucose oxidase, bee defensin-1). Gamma-irradiation sterilization procedure appears to be promising [74]. Also, it is important to agree on one standard method to determine the honey’s antimicrobial activity, making it easy to compare the activity of the product evaluated in different studies.

## 3. Antibacterial Activity of Bee Venom

Bee venom (BV) is a widely recognized toxin secreted by female worker bee’s poison glands as a protection mechanism [75]. Although it is toxic to predators, BV has been used for many medicinal purposes since Ancient Egypt (4000 BC). In bee venom therapy (BVT), the toxin is applied directly or indirectly into the body for the treatment of certain diseases, such as rheumatism arthritis [76,77,78]. Venoms from different animals or organisms are considered as promising antimicrobial agents that work against several bacteriological pathogenesis [79]. The antimicrobial activity and medical use of BV is due to the presence of bioactive molecules in particular peptides which are the main components and constitute 48–50% of dry BV weight [80]. The *Apis mellifera* venom is an odorless and transparent liquid that is made up of 88% of water and only 0.1 µg of dry venom [78]. The dry venom itself is highly rich in peptides notably melittin, apamin, adolapin, and mast cell degranulating (MCD) peptide, enzymes such as phospholipase A_2_ (PLA_2_) and hyaluronidas [81,82]. Among these peptides, melittin represents the main biological active compound in bee venom. It represents about 50% of the dry BV [83]. Another major component is phospholipase A_2_, being the most toxic bee venom peptide [84]. These two components can act synergically and damage the cell membrane [8]. Melittin possesses a low selectivity to the cell membrane and acts strongly on its lipids via the process of pore formation. This process causes the release of the cell cytoplasmic contents and leads to cell lysis [85]. This mode of action is most likely to be responsible of the antibacterial properties of BV [86]. Several studies have demonstrated the ability of BV to kill both Gram-negative and Gram-positive bacteria [87,88]. Indeed, the antibacterial activity of BV from *Apis mellifera* purebred as well as hybrid was tested against five bacterial strains. The results showed that BV displayed an antibacterial activity against all five bacterial strains. Other studies proved that BV can be used as a complementary antimicrobial agent against pathogenic bacteria even if it is collected by different methods (i.e., from the top of the frames or from under the frames with distance between the bottom board and the brood chamber) [89]. Additionally, the bacterium *Borrelia burgdorferi* is known to be the main cause of Lyme disease. Researchers found that both BV and melittin possess significant effects on all the morphological forms of *B. burgdorferi,* even on antibiotic resistant attached biofilms. However, antibiotics when administrated alone or combined together had limited effects [90]. Food-borne pathogens present a risk to develop antimicrobial resistance and generate biofilms such as *Salmonella*. Sixteen *Salmonella* strains isolated from poultry were tested against apitoxin (Bee venom). In 14 of the 16 tested strains, apitoxin reduced the biofilm formation and destroyed the preformed biofilm by 47%. Also, apitoxin increased the mobility of the bacteria and the MIC varied between 1024–256 μg/mL [7]. Aiming to find an alternative to antibiotics in the poultry industry, broiler chicks were sprayed with BV to evaluate its immunoprophylactic effects against *Salmonella gallinarum*. The results showed that BV increased the antibody production against formalin-killed *S. gallinarum* [91].

Others previous studies focused on the antibacterial effect of bee venom against additional type of bacteria such as *S. aureus* and *E. coli*. The results proved BV to be active in killing both bacterial strains. At 10 × MIC, the regrowth of *E. coli* was not observed at 18 h while For *S. aureus* at 5 × MIC [92,93]. Regarding the MIC for Gram-positive strains, the range was from 200 µg/mL to 8 µg/mL for the most sensitive species *B. subtilis*. Alternatively, Gram-negative strains were found to be more resistant to BV (MIC 60 to >500 µg/mL). This can be explained by the nature of the bacterium cell membrane [87,93]; the outer membrane of Gram-negative bacteria encompasses lipopolysaccharides (LPS) which obstruct the penetration of melittin presented in the BV. Thus, making melittin responsible for the antibacterial activity. While in Gram-positive bacteria, LPS are absent, indicating that melittin can penetrate the cell membrane more easily to form pores and increases the cell permeability through the cytoplasmic membrane [94]. PLA_2_, which is also presented in abundance in the crude BV can disrupt the cell membrane of Gram-negative bacteria indirectly by hydrolyzing phospholipids enzymatically. Other works reported the antibacterial activity of the crude BV and its two main biopeptides melittin and PLA_2_ against oral pathogen responsible of tooth decay: *Lactobacillus casei, Streptococcus salivarius, S. mutans, Staphylococcus mitis, Staphyslococcus sobrinus, Staphylococcus sanguinis* and *Enterococcus faecali*. Melittin was twice as active as the crude BV against the tested bacteria (4 to 40 µg/mL). As for PLA_2_, it was only active against *L. casei* at >400 µg/mL [95,96]. Similarly, melittin showed antimicrobial activity against Staphylococcal strains as well as MRSA strains while PLA_2_ did not show any effect on the same strains [97]. Also, it has been demonstrated that certain amino acids and their positions play a crucial role in the antibacterial activity exerted by melittin. For example, the absence of a proline residue in position 14 significantly diminishes the antimicrobial activity of melittin [98]. Likewise, two synthetic melittins, one asparagine-substituted melittin (Mel-N) and the other serine-substituted melittin (Mel-S) were able to penetrate the membrane of *E. coli* but the latter was more efficient [99]. Intriguingly, the antibacterial mechanism of new melittin derived-peptides MDP1 (GIGAVLKVLTTGLPALIKRKRQQ) and MDP2 (GIGAVLKWLPALIKRKRQQ) was evaluated against multidrug resistant strains such as *E. coli, P. aeruginosa* and MRSA. The two derived peptides showed strong antibacterial activity against reference strains of the three multidrug resistant strains compared to the indigenous melittin via membrane alteration and damage [100].

On the other hand, antimicrobial treatments are becoming less efficient by the day due to the increasing number of multi-drug resistant (MDR) bacteria as well as bacterial biofilms which are very hard to treat. Between 317 positive specimens for bacterial growth, 124 were MDR isolate of Gram-negative and Gram-positive bacteria. BV demonstrated a complete growth inhibition percentage (100%) against all tested MDR-isolates with a wide range of MICs and MLCs concentration-ranging between 3.125–50 μg/mL and 6.25–100 μg/mL, respectively against all MDR-GNB and GPB one [101]. The antibacterial activity of BV was tested alone or in combination with antibiotics (ampicillin, gentamicin, penicillin, or vancomycin) on the growth of MRSA strains. A partial synergetic effect was indicated by the index of inhibitory concentration ranging from 0.631 to 1.002 for the combination of BV with ampicillin or penicillin which made the MRSA strains more susceptible to the combination of BV with gentamicin or vancomycin rather than the combination of BV with ampicillin or penicillin [102]. BV was studied for its antibacterial activity against four MDR-*Acinetobacter baumannii* bacterial strains. Results showed an inhibition of the bacterial growth of MDR-*A. baumannii* strains with MIC value 31.25 mg/mL [103]. Melittin combined with doripenem caused a significant decrease in the MIC of MDR- *A. baumannii* strains. The combination of melittin–ceftazidime and melittin–doripenem was administrated to MDR-*P. aeruginosa* strains. This caused a decrease in melittin dosage which led to a decrease in melittin cytotoxicity. Therefore, the combination of melittin–doripenem (for *A. baumannii*) and melittin–doripenem with melittin– ceftazidime (for *P. aeruginosa*) at their MIC dose might serve as a promising therapeutical treatment against MDR bacteria [104].

The investigation of BV in pre-clinical and clinical application is still very slow despite its efficacity when combined with other drugs to treat different types of bacteria. Researchers found that BV and melittin combined with other antibiotics like vancomycin, oxacillin, and amikacin yielded fractional inhibitory concentration (FIC) indices fluctuating between 0.24 and 0.5. Apitoxin and melittin tested against 51 strains of bacteria exhibited strong antibacterial activity against both Gram-negative bacteria (MIC values between 30 and >500 μg/mL) and Gram-positive (MIC values between 10 and 100 μg/mL). Moreover, a strong anti-MRSA and anti-VRE activity was shown by BV and melittin at MIC values of 6–800 μg/mL [87]. When combined with oxacillin, BV and melittin showed an antibacterial activity against MRSA ATCC 3359 [84]. The antibacterial activity of melittin as well as its synergistic effect with *β*-lactam antibiotics against *A. baumannii* was evaluated by means of the broth microdilution method. The MIC value of melittin was 4 µg/mL and results showed inhibition of bacterial growth. Furthermore, FIC indices for combination of melittin with co-amoxiclav, ceftazidime and imipenem showed a synergetic effect [105].

All of the above- mentioned information confirm that BV and its two major compounds melittin and PLA_2_ exhibit a very promising antibacterial activity against several pathogenic bacterial strains even against multidrug resistant bacteria. However, one should always keep in mind that these biopeptides are venomous and might act as toxic agents if administrated the wrong way. Melittin is the most toxic component in BV. While being responsible for the majority of the pain associated with bee stings, it only induces a minor allergic reaction. Contrary to PLA_2_ which is the most allergenic and immunogenic protein in bee venom [106,107]. Bee envenomation can lead to many clinical manifestations such as local inflammatory reactions, allergic reactions, anaphylactic shocks, and systemic toxic reactions [108]. The ideal treatments against the toxic effect of bee venom are corticosteroids, antihistamine, and intramuscular adrenaline depending on the severity of the bee envenomation [109,110].

Many clinical studies on bee venom have shown promising results. Acne, one of the most common problems among teenagers, is usually treated with antibiotics to kill the causative bacteria but in some cases bacterial resistance can occur [111]. In a clinical study conducted by Han et al., skincare products with or without were tested on 12 subjects suffering from acne. The results showed a 57.7% decrease in ATP measured to evaluate the decrease in skin microbes. Thus, cosmetics and skincare products containing BV can used as a promising therapeutic anti-acne agent [112].

## 4. Antibacterial Activity of Propolis

Among the many products of honeybees, propolis is considered as one of the most interesting products utilized as the main defensive element and building block in the hives. Propolis is used by bees to fill the cavity in the walls of the hive, it is also used to repair combs and strengthen its thin borders. On the other hand, propolis can mummify intruders that cannot be transported outside thus preventing their decay [113]. Since ancient times, the anti-putrefactive property has been used by humans, notably by Egyptians. The origin of propolis, also known as “bee glue”, had long been a subject of discussion, some thought it came from bees themselves while others thought it originated from plants. Nowadays, the approximate composition of propolis and the factors affecting it became clearer [114]. Propolis is now confirmed to be a bee product made from plants. Bees collect resinous vegetable matter from different parts of the plant such as lipophilic matter on leaves and their buds, latex as well as mucilage [115]. Also, the bees are able to cut the fragments of vegetative tissues in order to extract the products necessary for the production of propolis [116]. Furthermore, propolis is a complex mixture with a variable composition that depends on the geographical region and the plant species used in its production. Surprisingly, having different composition does not indicate different biological activities [12]. Propolis is a resin that can occur in different colors and exhibits a pleasant aromatic resin smell of great value. Propolis is mainly composed of resins, flavonoids, polyphenols, terpenoids, essential oils, and other organics and minerals [117]. The extraction of propolis and its dissolution is needed in order to release its most active ingredients. The extraction process is also required for the later usage of propolis for medicinal purposes. It is also important to mention that solvent types used in extraction can affect the biological activity obtained from propolis. For the antibacterial activity, the abundancy of flavonoids and phenols plays a key role in conferring this bioactivity [118]. Additionally, the presence of many bioactive ingredients and in different concentrations is crucial in preventing the occurrence of bacterial resistance [119]. As for the mechanism of action, propolis can act directly on the microorganism or indirectly by triggering the immune system which results in the activation of the body natural defense mechanism. Studies showed that Gram-negative bacteria are more resistant to propolis than Gram-positive bacteria. This can be explained by two main factors, the first one being the specific structure of the Gram negative bacteria membrane and the second one being the secretion of hydrolytic enzymes which destroy the active ingredients present in propolis [120].

Artepillin C (3, 5-diprenyl-*p*-coumaric acid) is considered as one of the most important phenolic compounds found in propolis. Propolis ethanolic extract showed higher antibacterial activity against MRSA in comparison with hexane extract due to the higher concentration of Artepillin C in ethanolic extract [121]. The antibacterial activity of ethanol extracted propolis and its derivative compounds was investigated against *P. gingivalis*, a bacterium responsible for periodontal diseases. Artepillin C showed a bacteriostatic effect with membrane blebbing [122]. Propolis contains other phenyl derivatives such as 2-dimethyl-8-prenylchromene and 3-prenylcinnamic acid allyl ester. After investigating the chemical constituents and the antibacterial activity of the ethanolic extract of propolis, the results showed a high concentration of *p*-coumaric acid, artepillin-C, drupanin and kaempferide alongside an antibacterial activity against *Listeria monocytogenes, Enterococcus faecalis, S. aureus* and *Staphylococcus saprophyticus* [123]. Also, propolis contains pinocembrin and apigenin. Chilean propolis was subjected to a study and researchers found that the antibacterial activity of pinocembrin and apigenin is higher than that of polyphenols mixture or even chlorhexidine against *S. aureus* [124]. Numerous work reported the presence of antibacterial activity of pinocembrin against different bacterial strains such as *S. mutans, S. sobrinus, S. aureus, E. faecalis, L. monocytogenes, P. aeruginosa* and *K. pneumoniae* [125,126].

Nepalese ethanolic extract of propolis was evaluated for both its antimicrobial activity and its chemical composition. The main components were flavonoid aglycones (mainly neoflavonoids, isoflavonoids) and pterocarpans. Nepalese propolis exhibited its highest antibacterial activity against *H. pylori, S. aureus* and *Shigella flexneri*. When combined with amikacin and tetracycline, the same propolis yielded the strongest effect against *S. aureus* [126]. The ethanolic extract of polish propolis (EEPP) was examined for its antibacterial activity against MRSA and MSSA. The average MIC was 0.54 mg/mL and EEPP was effective against 12 *S. aureus* strains. Also, EEPP combined with 8 antistaphylococcal drugs, provided a stronger antibacterial effect against the tested strains than EEPP alone [127]. The geographical location affects the composition of propolis as well as its antimicrobial activity. 40 ethanol extract of propolis (EEP) collected worldwide were evaluated for their antibacterial activity against *S. aureus* strains. Results showed a moderate activity for Asian and African samples with MICS ranging from 0.0156 to >0.5 mg/mL and 0.0078 to >0.5 mg/mL, respectively with a similar results displayed by samples collected from North and South America and Europe [128]. EEP samples from three different regions of turkey were analyzed. MIC values varied and amounted to 0.018, 0.162, and 0.101 mg/mL and samples showed high antibacterial activity [129].

The number of studies concerning the antibacterial effect of propolis against anaerobic bacteria remains limited. However, it has been presented that propolis is more active on Gram-positive anaerobic bacteria than on Gram-negative ones. Propolis showed antibacterial activity against *Lactobacillus acidophilus, Prevotella oralis, P. gingivalis, Fusobacterium nucleatum, Peptostreptococcus anaerobius, Actinomyces naeslundii* and *Veillonella parvula* [130]. Moreover, research shows an important activity of propolis against *Porphyromona, Fusobacterium, Propionibacterium, Clostridium, Prevotella, Actinomyces* and *Bacteroides* species [131,132].

Propolis displays different antibacterial mechanisms such as inhibition of cell division, collapse of membranes and cell walls of the microbial cytoplasm, decreasing bacterial motility, enzymatic inactivation, bacteriolysis and inhibition of protein synthesis [133]. All the different compounds that constitute the propolis composition are rich in polyphenols which work together with different microbial proteins to form both ionic and hydrogenic bonds. This contributes to the modification of the three-dimensional structure of proteins hence the alteration of their function. These effects have encouraged researchers to combine propolis with antibiotics in order to overcome the problem of bacterial resistance to drugs [134]. A synergy has been observed between EEP, vancomycin and oxacillin; antibiotics that inhibit the synthesis of the bacterial cell wall, against *S. pyogenes*, VRE ATCC 51299 and MRSA NCTC 10442 [135]. Also, synergism was present between EEP and drugs targeting microbial ribosomes (neomycin) but absent with drugs interfering in the biosynthesis of folic acid or DNA (ciprofloxacin and norfloxacin) as well as those inhibiting metabolic pathways (cotrimoxazole) [134]. EEP has proved to be the most effective when combined with antibiotics that interfere with bacterial protein biosynthesis such as tetracycline, linezolid, chloramphenicol, gentamicin, tobramycin and netilmicin against MRSA and MSSA [127].

Propolis is widely used in medicine due to its various bioactivities. The effect of topical propolis extract was tested on facial vulgaris acne where subjects were treated with a topical solution of ethanolic extract of propolis. This solution showed a significant bacteriological activity on *Propionibacterium acnes* and *Staphylococcus epidermidis*, as well as being effective against facial acne [136]. To test the efficiency of propolis in inhibiting dental caries, propolis fluoride was applied to children’s teeth with active dentinal carries surface. Carries were stopped in 99.80% of the cases after just one month of application without causing any black discoloration of the teeth [137]. In another study, researchers treated patients suffering from chronic periodontitis with an irrigation of the selected sites with a hydro alcoholic solution of propolis extract propolis. Results showed that propolis exhibits a considerable activity against chronic periodontitis. This treatment was more effective than the conventional treatment as it significantly decreased the viability of *P. gingivalis* [138].

## 5. Antibacterial Activity of Bee Pollen

Pollen of various flowers is collected by field bees and agglutinated by bees enzymes secreted by the salivary glands and by the nectar. Then, the pollen is placed in corbiculae situated on the tibia of the bees hind legs to form the pollen loads, commonly known as the bee pollen, in the form of granules [139]. Pollen is the raw material used by bees to produce bee bread, the basis hives food [140]. Pollen stored in honeycomb cells is covered with a thin layer of honey and wax. This results in the formation of bee bread which undergoes anaerobic fermentation and is preserved by the lactic acid which formed [141]. In comparison to honey, small amounts of bee pollen are stocked in the hives and are used during forage absence [142]. Many studies have shown that bees collect pollen from very few plant species. Bee pollen contains about 250 substances including amino acids, lipids, vitamins, macro- and micronutrients, flavonoid, and organic carotenoid pigments which makes it an ideal food [143]. Its composition varies depending on the biogeographic origin, ecological habitat, season, weather conditions during collection, as well as on the bee race and even beekeeping management [144]. Phenolic compounds are responsible of the bee pollen bioactivity [141]. For centuries, bee pollen has been used for its medicinal and nutritional properties. Likewise, many ancient philosophers highly valued pollen as a part of their healthy diet [15].

Bee pollen is often collected by using a pollen trap at the entrance or in the hives. Sometimes, it is directly collected from the bee hind legs [145]. Water, ethanol and methanol are the most commonly used solvents for the extraction of bee pollen. Studies showed that the highest content of bioactive compounds is present in ethanol or water extracts in comparison to natural bee pollen [146]. The antibacterial activity of bee pollen was investigated and results showed a positive effect on both Gram-positive and Gram-negative bacteria with a higher sensitivity in Gram positive bacteria. A study on the antibacterial activity of Moroccan bee pollen demonstrated an inhibition effect against *S. aureus, Streptococcus spp, E. coli* and *P. aeruginosa* with a higher sensitivity in Gram-positive bacteria [145]. Similarly, *Bacillus cereus, S. aureus, S. typhi* and *E. coli* were sensitive to Portugal bee pollen [147]. Applying a concentration of 0.02% to 2.5% (vol/vol) of bee pollen extract to *B. cereus, B. subtilis, E. coli, Salmonella typhimurium, S. aureus, Yersinia enterocolitica, E. faecalis* and *Listeria monocytogenes* did not exert any antimicrobial effect on these bacterial strains indicating that the activity is concentration dependent [148]. The antimicrobial activity of bee pollen was also assessed on Clostridia class organisms, i.e., *Clostridium butyricum, Clostridium hystoliticum, Clostridium intestinale, Clostridium perfringens and Clostridium ramosum*. Results showed an antibacterial activity against all of the above mentioned strains of clostridia with that against *C. butyricum* and *C. perfringens* being the most potent one [149]. The antimicrobial activity of a Chilean bee pollen extract was investigated against human pathogenic bacteria *E. coli, P. aeruginosa, S. aureus* and *S. pyogenes* showed a high sensitivity to the bioactivity of bee pollen in opposition to *E. coli* and *P. aeruginosa* [150].

Furthermore, ethanol and methanol extracts of Slovakian bee pollen were tested for their antibacterial property against *L. monocytogenes, P. aeruginosa, S. aureus, S. enterica* and *E. coli*. Results showed that *S. aureus* was the most sensitive bacteria to the ethanolic bee pollen extract while *S. enterica* was the most sensitive one to the methanolic extract [139]. *E. coli, S. enteritidis, S. aureus, L. monocytogenes* were tested with ethanol pollen extract. It was found that *S. aureus* and *L. monocytogenes* growth was inhibited while this was not the case for both *E. coli* and *Salmonella enteritidis* which both showed resistance to ethanol extracts of pollen [151]. In some studies, the nature of the solvent used might affect the antibacterial activity of bee pollen where the same sample can be more effective against Gram-negative bacteria than Gram-positive bacteria. Slovenian bee pollen ethanol extract showed a higher antibacterial activity against Gram-negative bacteria (*E. coli* and *Campylobacter jejuni*) than Gram-positive bacteria (*L. monocytogenes*) [152]. The same result was observed for honeybee pollen collected from a region of Chile who revealed antibacterial activity against *S. pyogenes* only and showed no activity against *E. coli, S. aureus* and *P. aeruginosa* [14]. Moreover, *E. coli* was the most sensitive strain to 70% ethanol bee pollen extract but resistant to 96% ethanol extract which confirms the fact that bacterial strains are specific to the solvent and its concentration [153].

The antibacterial mechanism of bee pollen remains unclear until now. Studies suggest that the antibacterial activity of bee pollen is associated with glucose oxidase; an enzyme produced by honeybees and added to pollen during the process of granules formation [154]. Additionally, this activity might be correlated with the total phenolic content and phenol composition. Phenolic acids and flavonoids present in pollen can act against bacterial cells by degrading the cytoplasmic membrane which leads to loss of potassium ions and initiation of the cell’s autolysis [148]. Other researchers argue that the composition of the pollen is associated with its antibacterial activity. This is most likely due to the fact that many bee pollen extracts with the lowest total phenol concentrations were the most effective against microorganisms [155,156]. In addition to the presence of many bioactive compounds such as fatty acids, EXLVs and presumably microbial metabolites known for their antibacterial activity in bee pollen. Surprisingly, bee pollen exerts an inhibition effect against many pathogenic bacterial strains but does not act against lactic acid starter cultures. Hence, bee pollen could serve as a potential candidate that is more suitable than antibiotics which kill both pathogens and probiotics [157].

## 6. Antibacterial Activity of Royal Jelly

Royal jelly (RJ), a yellowish white, sticky acidic secretion, is produced by worker bees and secreted from hypopharyngeal and mandibular glands. Bee larvae at early stages of their life rely on royal jelly as a primary source of food. Only the queen bee is fed by royal jelly until it dies, that’s why royal jelly is widely recognized as a ‘’super food’’ [158,159].

Water is the major molecule forming RJ (50 to 60%). In addition to water, RJ contains other components such as proteins (18%), carbohydrates (15%), lipids (3 to 6%), mineral salts (1.5%), and other minor constituents such as free amino acids, vitamins (particularly vitamin B) and phenols such as flavonoids [160].

RJ exhibits important antibacterial activity. This antibacterial potential of RJ is due to the existence of some main bioactive constituents. Proteins play an important role in the antibacterial activity of RJ. In fact, proteins are the major constituents of the dry matter of RJ (50% of the dry matter of RJ) [161,162,163,164]. The MRJPs glycoproteins family form 82–90% of the protein constitution in RJ and has nine identified members MRJP1-MRJP9 [165]. MRJP1, also known as royalactin [166] or apalbumin [167], is the first identified and major protein in the MRJPs family [165]. It has been discovered that MRJP1 possesses an indirect antibacterial activity. Moreover, there are jelleines, short antimicrobial peptides (eight to nine amino acids), which have four different peptide sequences: jelleine-I (PFKLSLHL-NH_2_), jelleine-II (TPFKLSLHL-NH_2_), jelleine-III (EPFKLSLHL-NH_2_), and jelleine-IV (TPFKLSLH-NH_2_). Jelleine I, II, and III are derived from MRJP1 cleavage product and are responsible for the antibacterial activity of MRJP1 [168,169]. A previous study has tested jelleines I, II and III against Gram-positive (*S. aureus, S. saprophyticus* and *B. subtilis*) and Gram-negative bacteria (*E. coli, Enterobacter cloacae, K. pneumoniae* and *P. aeruginosa*). The results revealed that jelleines-I and II had a broad-spectrum activity, while jelleine-III was less active, and jelleine-IV had no antimicrobial activity [168]. Hence, jelleines I, II and III are responsible for the antibacterial activity of MRJP1. On another hand, MRJP2 (apalbumin2) exhibited an antibacterial activity against *P. larvae, B. subtilis* and *E. coli*. This antibacterial activity of MRJP2 is attributable to its high mannose carbohydrate levels and its complex-type antennary carbohydrate structures [170]. In addition, a study has demonstrated that the levels of MRJP3 (apalbumin 3) increased after bacterial infection. Future work should investigate the antimicrobial activity of this protein against a variety of pathogens [171]. It is important to mention that the dominant allergens of royal jelly are MRJ1 and MRJ2, which could present a limitation to the usage of royal jelly as a therapeutic agent [172].

Royalisin, another antibacterial protein found in RJ, prevents RJ from becoing contaminated and infected by Gram-positive bacteria. Royalisin has been shown to have antibacterial activity against Gram-positive bacteria but not Gram-negative bacteria [168]. However, it has been demonstrated that royalisin plays a role in the honeybee protection system against bacterial invasion especially against *P. larvae*, the main pathogen of American foulbrood disease. Royalisin also acts on other Gram-positive bacteria like *Staphylococcus, Streptococcus, B. subtilis, Sarcina lutea, Micrococcus luteus* and Gram-positive rods such as *Corynebacterium, Clostridium, Lactobacilus helveticus* and *Leuconostoc.* However, no inhibition of Gram-negative bacteria like *E. coli* and *Serratia marcescens* was detected [173,174,175].

A study was conducted with the aim to assess the amount of royalisin in RJ obtained from honeybee colonies with different genotypes. It was found that there is an obvious link between the amount of royalisin and the antibacterial potential of RJ. Furthermore, the amount of royalisin in RJ was discovered to differ between colonies [176].

The mechanism of action of the majority of RJ peptides remains unrecognized. For the first time, the analysis of RAcc-Royalisin (recombinant protein RAcc-Royalisin expressed and purified from *E. coli*) showed that royalisin inhibits bacterial growth by disrupting the permeability of the cell membrane as well as reducing the hydrophobicity of bacterial cells [177].

Moreover, *trans*-10-hydroxy-2-decenoic acid (10-HDA) fatty acid, is the most abundant component in the lipids fraction (80%) and can only be found in RJ [178]. Unlike other acids, which have 14–20 carbon atoms, RJ fatty acids are shorter (8–10 carbon atoms). Furthermore, RJ fatty acids are either dicarboxylic or hydroxy acids. Other fatty acids, on the other hand, are normally triglyceride fatty acids [179]. Thus, this particular fatty acid may be used to distinguish RJ from other bee products and to confirm the authenticity of former [180]. Early studies on 10-HDA antimicrobial activity found that 10-HDA has a high capability to inhibit the growth of both Gram-negative bacteria (*E. coli)* and Gram-positive (*Micrococcus pyogenes, B. subtilis, S. aureus*) [181]. Also, a comparative study of the antimicrobial capacity of the ether-soluble fatty acids, which primarily contain 10-HDA, and the ether-insoluble fractions, which include royalisin, was also performed. Several pathogenic bacteria were tested to see if these fractions had antibacterial properties against strains of *Streptomyces species, S. aureus* and *E. coli*. The results show that at concentrations of 30 mg/mL, ether-soluble fractions of RJ strongly reduced the growth of the previously described microorganisms, while non-soluble fractions had almost no antimicrobial activity [182]. Furthermore, glucosyltransferases (gtfs) genes, especially gtfB and gtfC, are essential in *S. mutans* colonization and pathogenesis. Yousefi et al. discovered that 10-HDA inhibits gene expression and mRNA transcription while also effectively reducing *S. mutans* adhesion to the cell surface [183].

Milliou and Chinou reported other fatty acids that displayed an antibacterial activity. Oral pathogens *S. mutans* and *Streptococcus viridans*, as well as *S. aureus* and *S. epidermidis*, were efficiently inhibited by 3-hydroxydodecanedioic acid, 10-acetoxy-2-decenoic acid, and (11S)-hydroxydodecanoic acid. However, most of these fatty acids occurred to be ineffective against Gram-negative bacteria. Moreover, 3-hydroxydodecanedioic acid, with a MIC of 0.47 mg/mL, appeared to have a minor effect on *K. pneumonia* [184].

Consequently, some of MRJPs, jelleines, royalisin and 10-HAD may act synergically and confer the RJ an effective and promising antibacterial activity.

Raw RJ has been tested against human pathogens in many studies. Two RJ samples, from Argentina, A and B were tested against Gram-positive and Gram-negative bacteria that cause infections in cutaneous wounds. Both samples had the ability to inhibit Gram-positive and Gram-negative bacteria except for *K. pneumoniae* which was not inhibited by neither samples. Sample A also had no effect on *Streptococcus uberis* [185]. Differences in activities between samples of RJ could be linked to the differences in geographical locations and changes in components due to the genetic variations between bee colonies [185]. Another study has tested RJ against Gram-positive (*M. luteus, B. cereus* and *S. aureus*) and Gram-negative bacteria (*S. flexneri, S. typhi, E. coli, P. vulgaris* and *P. aeruginosa*). Results demonstrated that RJ exhibited an antibacterial activity and had two distinct forms of effect, one being bactericidal and the other being bacteriostatic against the studied bacteria. Likewise, they investigated the impact of storage time on RJ antibacterial activity. Within 24 h of being frozen, the highest antibacterial activity of RJ was observed. This activity decreased gradually over time until it reached a steady value [186]. A recent study has compared the activity of RJ to chlorhexidine (gold standard) against the periodontopathic bacteria (aerobic and anaerobic) in subgingival plaque. It was found that chlorhexidine has a greater inhibitory effect than RJ. These results indicated that RJ should be administrated at high concentrations if it was to be used as an alternative to chlorhexidine [187].

The combination of RJ with other compounds was also studied in order to enhance the effectiveness of RJ antibacterial activity. Boukraa et al. investigated the effect of mixing RJ with starch. They found that starch alone does not have an antibacterial activity. RJ MIC against *S. aureus* and *E. coli* was 1.7% (*v/v*) and 2% (*v/v*), respectively. When mixed with starch, the MIC decreased by 61% and 30% against *S. aureus* and *E. coli*, respectively. It was suggested that this decrease in MIC values is due to the existence of amylases in RJ which might be a possible explanation for the increase in the antibacterial activity after adding starch. This enzyme breaks down starch into dextrin and maltose, increasing RJ osmotic capacity and, as a consequence, its antibacterial impact [188]. Surprisingly, when RJ was mixed with honey, which is known to have a strong antibacterial activity, it was found that MIC values decreased significantly against *P. aeruginosa* [189].

Furthermore, Romanelli et al. found that when jelleine-I was combined with temporin A or temporin B (peptides from the temporin family that are active against Gram-positive bacteria at low concentrations), the antibacterial activity of jelleine-I was increased against *S. aureus* and *L. monocytogenes* [190].

## 7. Conclusions

The use of beehive products for medicinal purposes has long been exploited by mankind. Here, the antibacterial properties of honey, bee venom, propolis, pollen and royal jelly have been discussed. Each of these products is characterized by the presence of bioactive compounds which makes the formers powerful inhibitors of pathogenic bacterial strains. Table 1 summarizes all the bacterial strains susceptible to honeybee products with the MIC and MBC determined. Many clinical trials are being conducted to test the safety and effectiveness of beehive products on infected wounds as well as on various bacterial diseases such as gastroenteritis. Studies showed that bee venom exhibits both antibacterial and anti-inflammatory activity and can be used in acne treatment, and propolis was demonstrated to be efficient in both skin and dental treatment. Finally, although bee products are considered as promising and potent antibacterial candidates, future work should emphasize on their possible adverse effects when applied or administrated directly to the human body.

## Figures and Tables

**Table 1:** Summary of all the bacterial strains susceptible to each honeybee product. The minimum inhibitory effect (MIC) as well as the minimum bactericidal concentration (MBC) of each of the beehive products are also shown (ND: none determined).

Honeybee Product			Bacterial Strains	MIC/MBC	Reference
**Honey**	**Raw honey**	Manuka honey	*Shigella sonnei*	MIC 9%(*v/v*)	[191]
		Talah honey		MIC 20%(*v/v*)	
		Saudi honey	*S. flexneri*	MIC 10–20%(*v/v*)	[192]
		Canadian honey	*E. coli*	MIC 6.25% (*w/v*) MBC 6.25–12.5%(*w/v*)	[44]
		Saudi honey		MIC 20–40%(*v/v*)	[192]
		Korean honey		MIC 25–50%(*w/v*)	[193]
		Omani and African honey		ND	[194]
		Ulmo honey		MIC 12.5%(*v/v*)	[195]
		Manuka honey	MRSA	MIC 9.98%(*v/v*)	[46]
		Pasture honey		MIC 3.07%(*v/v*)	
		Ulmo honey		MIC 3.1–6.3%(*v/v*)	[195]
		Turkish honey	*S. aureus*	ND	[196]
		Saudi honey		MIC 20–40%(*v/v*)	[192]
		Brazilian honey		MIC 126.23–185.70 mg.mL^−1^	[197]
		Korean honey		MIC 25–50%(*w/v*)	[193]
		Omani and African honey		ND	[194]
		Algerian honey		MIC 12–95%(*v/v*)	[55]
		Saudi honey	*Staphylococcus epidermis*	MIC 20–40%(*v/v*)	[192]
		Manuka honey	vancomycin-sensitive *Enterococci*	MIC 4.92%(*v/v*)	[46]
		Pasture honey		MIC 9.66%(*v/v*)	
		Manuka honey	vancomycin-resistant *Enterococci*	MIC 4.61%(*v/v*)	
		Pasture honey		MIC 8.25%(*v/v*)	
		Manuka honey	Coagulase-negative *staphylococci*	MIC 3.4%(*v/v*)	[198]
		Pasture honey		MIC 3.6%(*v/v*)	
		Manuka honey	*Campylobacter spp.*	MIC ≈ 1% (*v/v*)	[199]
		Turkish honey	*H. pylori*	ND	[196]
		Cider honey			[48]
		Turkish honey	*B. subtilis*	ND	[196]
		Saudi honey	*Proteus mirabilis*	MIC 10–20%(*v/v*)	[192]
		Korean honey	*L. monocytogenes*	MIC 25% (*w/v*)	[193]
		Korean honey	*Salmonella typhymurium*	MIC 25–50% (*w/v*)	
		Omani honey	*P. aeruginosa*	ND	[194]
		African honey			
		Ulmo honey		MIC 12.5% (*v/v*)	[195]
		Tualang honey		MIC 18.5% (*w/v*) MBC 25% (*w/v*)	[200]
		Manuka honey		MIC 7.5%(*v/v*) MBC 9.71%(*v/v*)	[201]
		Pasture honey		MIC 6.8%(*v/v*) MBC 9%(*v/v*)	
		Tualang honey	*S. pyogenes*	MIC 13% (*w/v*) MBC 25% (*w/v*)	[200]
		Algrian honey		25–73%(*v/v*)	[55]
		Manuka honey	*S. mutans*	MIC 100–200 μg/mL MBC 200–500 μg/mL	[51]
		Manuka honey	*B. cepacia*	MIC 2.9%(*v/v*)	[53]
		Pasture honey		MIC 3.6%(*v/v*)	
			*P. gingivalis*	MIC 2–10% (*w/v*)	[57]
	Hydrogen peroxide	Melilot honey (sample 5)	*S. aureus*	MIC 12.5% (*w/v*)	[32]
		Dutch Gold Honey		ND	[33]
		Dutch Gold honey	*S. sonnei*	ND	[33]
		Melilot honey (sample 5)	*Salmonella spp.*	MIC 12.5% (*w/v*)	[32]
		Melilot honey (sample 5)	*E. coli*	MIC 12.5% (*w/v*)	[32]
		Dutch Gold honey	*L. monocytogenes*	ND	[33]
	Methylglyoxal	Manuka honey	*B. subtilis*	MIC 0.8 mM	[202]
			*S. aureus*	MIC 1.2 mM	[202]
			*P. aeruginosa*	MIC 1.0 mM	[202]
			*E. coli*	MIC 1.2 mM	[202]
**Bee venom**	**Crude extract**		MRSA CCARM 3366	MIC 0.085 μg/mL MBC 0.106 μg/ mL	[102]
			MRSA ATCC 33591	MIC_90%_ 7.2 μg/mL MBC_90%_ 28.7 μg/mL	[84]
			*S. aureus* CCARM 3708	MIC 0.11 μg/mL MBC 0.14 μg/mL	[102]
			*S. aureus* enterotoxin ATCC 23235	MIC 0.7 μg/mL	[84]
			*S. salivarius*	MIC 20 µg/mL	[95]
			*S. sanguinis*	MIC 30 µg/mL	
			*S. sobrinus*	MIC 40 µg/mL	
			*S. mitis*	MIC 40 µg/mL	
			*S. mutans*	MIC 20 µg/mL	
			*K. pneumonia*	MIC 30 µg/mL for 24 h	[87]
			*B. subtilis*		
			*E. faecalis*	MIC 20 µg/mL MIC 3.7 µg/mL	[95]
			*L. casei*	MIC 20 µg/mL MIC 0.9 µg/mL	
			*Borrelia spirochetes*	MIC 200 µg/mL MIC 10 µg/mL	[90]
			*E. coli*	MIC 0.25 µg/mL	[92]
			*S. aureus*	MIC 0.06 µg/mL	
			*S. salivarius*	MIC 10 µg/mL	[95]
			*E. FAECALIS*	MIC 6 µg/mL	
			*L. casei*	MIC 4 µg/mL	
	**Melittin**		*S. sanguinis*	MIC 10 µg/mL	
			*S. sobrinus*		
			*S. mitis*		
			*S. mutans*	MIC 40 µg/mL	
			*K. pneumonia*	MIC 8 µg/mL throughout 24 h	[87]
			*B. subtilis*	MIC 6 µg/mL for 24 h	
			*L. monocytogenes* F4244	MIC 0.315 µg/mL MBC 3.263 µg/mL	[94]
			*E. coli*	MIC 0.125 µg/mL	[92]
			*S. aureus*	MIC 0.06 µg/mL	
			MRSA ATCC 33591	MIC_90%_ 6.7 μg/mL MBC_90%_ 26 μg/mL	[84]
			*S. aureus* enterotoxin ATCC 23235	MIC 3.6 μg/mL	[84]
			*B. spirochetes*	MIC 200 µg/mL	[90]
	**Mutant melittin I17K**		*L. monocytogenes F4244*	MIC 0.814 µg/mL MBC 7.412 µg/mL	[94]
	**Mutant melittin G1I**			MIC 0.494 µg/mL MBC 5.366 µg/mL	
	**PLA2**		*L. casei*	MIC 400 µg/mL	[95]
**Propolis**			*S. aureus*	MIC_90_ 246.3 μg/mL	[121]
			*P. gingivalis* ATCC 33277	MIC 64–128 μg/mL	[122]
			*S. aureus* ATCC 25923	MIC 6.2 mg/mL	[123]
			*L. monocytogenes*		
			*E. faecalis* ATCC 19433		
			*S. saprophyticus* ATCC 15305		
			*S. aureus* (ATCC 25923)	MIC 0.39 mg/mL	[125]
			*E. faecalis* (ATCC 29212)		
			*E. coli* (ATCC 25922)		
			*P. aeruginosa* (ATCC 27853)		
			*L. monocytogenes* (ATCC, 19111)	MIC 0.10 mg/mL	
			*K. pneumonia* (ATCC 13883)	MIC 0.78 mg/mL	
			*S. aureus*	MIC_50_ 0.39 mg/mL MIC_90_ 0.78 mg/mL MBC 0.78 to 3.13 mg/mL	[127]
			*S. typhi*	MIC 9.90% *v/v* MIC 10.0% *v/v*	[134]
**Royal jelly**			*S. aureus*	MIC 3.4–9 mg/mL MBC < 250 mg/mL	[185]
				MIC 12.5 mg/mL	[186]
				MIC 1.7% (*v/v*)	[188]
			*MRSA*	MIC 8–14.5 mg/mL MBC < 250 mg/mL	[185]
			*E. coli*	MIC 13.5 mg/mL	[186]
				MIC 2% (*v/v*)	[188]
			*S. epidermis*	MIC 8.7–10.3 mg/mL MBC 125 mg/mL	[185]
			*M. luteus*	MIC 7.5–11.8 mg/mL MBC 125 mg/mL	
			*E. faecalis*	MIC 3.7–17.7 mg/mL MBC < 250mg/mL	
			*P. aeruginosa*	MIC 3.3–14.4 mg/mL	
				MIC 15.5 mg/mL	[186]
			*P. vulgaris*	MIC 15.5 mg/mL	
			*S. typhi*	MIC 14.5 mg/mL	
			*B. cereus*	MIC 12.5 mg/mL	
			*Sarcina lutia*	MIC 0.30 mg/mL	
			*S. flexneri*	MIC 14.5 mg/mL	
**Pollen**			*B. cereus*	MIC 0.17% (*w/v*)	[147]
			*S. aureus*	MIC 0.21% (*w/v*)	
			*E. coli*	MIC 82.4 mg/mL	[150]
			*P. aeruginosa*	MIC 41.2 mg/mL	
			*S. aureus pyogenes*	MIC 20.6 mg/mL	
			*E. coli*	MIC 2.68 mg/mL	[152]
			*C. jejuni*	MIC 9.93 mg/mL	
			*L. monocytogenes*	MIC > 6.25 mg/m	
				MIC 320 µg/mL	[157]
			*E. coli*		
			*S. enteritidis*		
			*S.* *aureus*		
			*S. pyogenes*	MIC 0.78–6.25 mg/mL	[14]
			*P. aeruginosa*	MIC 640 µg/mL	[157]

## Data Availability

Not applicable.

## References

[1] D’Apolito Pessoa S.M., Balestieri F.C., de LM Balestieri J.B.P. (2010). Pollen harvest by Apis mellifera L. (Hymenoptera: Apidae) in the Dourados region, Mato Grosso do Sul state (Brazil). Acta Bot. Bras..

[2] Greenleaf S.S., Kremen C. (2006). Wild bees enhance honey bees’ pollination of hybrid sunflower. Proc. Natl. Acad. Sci. USA.

[3] Bogdanov S. (2016). Pollen: Nutrition, Functional Properties, Health. Bee Prod. Sci..

[4] Kuropatnicki A.K., Szliszka E., Krol W. (2013). Historical aspects of propolis research in modern times. Evid Based Complement. Altern. Med..

[5] Kassim M., Achoui M., Mansor M., Yusoff K.M. (2010). The inhibitory effects of Gelam honey and its extracts on nitric oxide and prostaglandin E2 in inflammatory tissues. Fitoterapia.

[6] Khalil W.K.B., Assaf N., ElShebiney S.A., Salem N.A. (2015). Neuroprotective effects of bee venom acupuncture therapy against rotenone-induced oxidative stress and apoptosis. Neurochem. Int..

[7] Arteaga V., Lamas A., Regal P., Vazquez B., Miranda J., Cepeda A., Franco C.M. (2019). Antimicrobial activity of apitoxin from Apis mellifera in Salmonella enterica strains isolated from poultry and its effects on motility, biofilm formation and gene expression. Microb. Pathog..

[8] Abd El-Wahed A.A., Khalifa S.A.M., Sheikh B.Y., Farag M.A., Saeed A., Larik F.A., Rahman A.U. (2019). Chapter 13 Bee Venom Composition: From Chemistry to Biological Activity. Studies in Natural Products Chemistry.

[9] Yaacoub C., Rifi M., El-Obeid D., Mawlawi H., Sabatier J.-M., Coutard B., Fajloun Z. (2021). The Cytotoxic Effect of Apis mellifera Venom with a Synergistic Potential of Its Two Main Components-Melittin and PLA2-On Colon Cancer HCT116 Cell Lines. Molecules.

[10] Cornara L., Biagi M., Xiao J., Burlando B. (2017). Therapeutic Properties of Bioactive Compounds from Different Honeybee Products. Front Pharmacol. https://www.frontiersin.org/articles/10.3389/fphar.2017.00412/full.

[11] Paulino N., Abreu S.R.L., Uto Y., Koyama D., Nagasawa H., Hori H., Koca-Caliskan U., AlAjmi M.F., Hassan M., Wahabi H.A. (2008). Anti-inflammatory effects of a bioavailable compound, Artepillin C, in Brazilian propolis. Eur. J. Pharmacol..

[12] Schnitzler P., Neuner A., Nolkemper S., Zundel C., Nowack H., Sensch K.H., Reichling J. (2010). Antiviral activity and mode of action of propolis extracts and selected compounds. Phytother. Res..

[13] Buchwald R., Breed M.D., Greenberg A.R., Otis G. (2006). Interspecific variation in beeswax as a biological construction material. J. Exp. Biol..

[14] Bridi R., Atala E., Pizarro P.N., Montenegro G. (2019). Honeybee Pollen Load: Phenolic Composition and Antimicrobial Activity and Antioxidant Capacity. J. Nat. Prod..

[15] Campos M., Frigerio C., Lopes J., Bogdanov S. (2010). What is the future of Bee-Pollen?. J. ApiProduct ApiMedical Sci..

[16] Ahmed R., Tanvir E.M., Hossen M.S., Afroz R., Ahmmed I., Rumpa N.-E.-N., Paul S., Gan S.H., Sulaiman S.A., Khalil M.I. (2017). Antioxidant Properties and Cardioprotective Mechanism of Malaysian Propolis in Rats. Evid. Based Complementary Altern. Med..

[17] Inoue S., Koya-Miyata S., Ushio S., Iwaki K., Ikeda M., Kurimoto M. (2003). Royal Jelly prolongs the life span of C3H/HeJ mice: Correlation with reduced DNA damage. Exp. Gerontol..

[18] Simúth J., Bíliková K., Kovácová E., Kuzmová Z., Schroder W. (2004). Immunochemical approach to detection of adulteration in honey: Physiologically active royal jelly protein stimulating TNF-alpha release is a regular component of honey. J. Agric. Food Chem..

[19] Nakaya M., Onda H., Sasaki K., Yukiyoshi A., Tachibana H., Yamada K. (2007). Effect of Royal Jelly on Bisphenol A-Induced Proliferation of Human Breast Cancer Cells. Biosci. Biotechnol. Biochem..

[20] Kohno K., Okamoto I., Sano O., Arai N., Iwaki K., Ikeda M., Kurimoto M. (2004). Royal jelly inhibits the production of proinflammatory cytokines by activated macrophages. Biosci. Biotechnol. Biochem..

[21] Hattori N., Ohta S., Sakamoto T., Mishima S., Furukawa S. (2011). Royal jelly facilitates restoration of the cognitive ability in trimethyltin-intoxicated mice. Evid Based Complement Altern. Med..

[22] Blasa M., Candiracci M., Accorsi A., Piacentini M., Albertini M., Piatti E. (2006). Raw Milefiori honey is packed full of antioxidant. Food Chem..

[23] Ahmed A.K.J., Hoekstra M.J., Hage J.J., Kariml R.B. (2003). Honey-medicated dressing: Transformation of an ancient remedy into modern therapy. Ann. Plast. Surg..

[24] Moniruzzaman M., Khalil M.I., Sulaiman S.A., Gan S.H. (2013). Physicochemical and antioxidant properties of Malaysian honeys produced by Apis cerana, Apis dorsata and Apis mellifera. BMC Complementary Altern. Med..

[25] Khalil M., Moniruzzaman M., Boukraâ L., Benhanifia M., Islam M., Sulaiman S.A., Gan S.H. (2012). Physicochemical and Antioxidant Properties of Algerian Honey. Molecules.

[26] Brown E., O’Brien M., Georges K., Suepaul S. (2020). Physical Characteristics and Antimicrobial Properties of Apis Mellifera, Frieseomelitta Nigra and Melipona Favosa Bee Honeys from Apiaries in Trinidad and Tobago. BMC Complement Med. Ther..

[27] Gheldof N., Wang X.-H., Engeseth N.J. (2002). Identification and quantification of antioxidant components of honeys from various floral sources. J. Agric. Food Chem..

[28] Da CAzeredo L., Azeredo M.A., De Souza S.R., Dutra V.M. (2003). Protein contents and physicochemical properties in honey samples of Apis mellifera of different floral origins. Food Chem..

[29] Kumar P., Sindhu R.K., Narayan S., Singh I. (2010). Honey collected from different floras of Chandigarh Tricity: A comparative study involving physicochemical parameters and biochemical activities. J. Diet Suppl..

[30] Kuś P.M., Szweda P., Jerković I., Tuberoso C.I.G. (2016). Activity of Polish unifloral honeys against pathogenic bacteria and its correlation with colour, phenolic content, antioxidant capacity and other parameters. Lett. Appl. Microbiol..

[31] Bang L.M., Buntting C., Molan P. (2003). The effect of dilution on the rate of hydrogen peroxide production in honey and its implications for wound healing. J. Altern. Complement Med..

[32] Sowa P., Grabek-Lejko D., Wesołowska M., Swacha S., Dżugan M. (2017). Hydrogen peroxide-dependent antibacterial action of Melilotus albus honey. Lett. Appl. Microbiol..

[33] Taormina P.J., Niemira B.A., Beuchat L.R. (2001). Inhibitory activity of honey against foodborne pathogens as influenced by the presence of hydrogen peroxide and level of antioxidant power. Int. J. Food Microbiol..

[34] Brudzynski K., Abubaker K., Miotto D. (2012). Unraveling a mechanism of honey antibacterial action: Polyphenol/H₂O₂-induced oxidative effect on bacterial cell growth and on DNA degradation. Food Chem..

[35] Abdulrhman M.A., Mekawy M.A., Awadalla M.M., Mohamed A.H. (2010). Bee honey added to the oral rehydration solution in treatment of gastroenteritis in infants and children. J. Med. Food.

[36] Ratiu I.A., Al-Suod H., Bukowska M., Ligor M., Buszewski B. (2019). Correlation Study of Honey Regarding their Physicochemical Properties and Sugars and Cyclitols Content. Molecules.

[37] Kwakman P.H.S., te Velde A.A., de Boer L., Speijer D. (2010). Vandenbroucke-Grauls CMJE, Zaat SAJ. How honey kills bacteria. FASEB J..

[38] Adams C.J., Manley-Harris M., Molan P.C. (2009). The origin of methylglyoxal in New Zealand manuka (Leptospermum scoparium) honey. Carbohydr. Res..

[39] Mavric E., Wittmann S., Barth G., Henle T. (2008). Identification and quantification of methylglyoxal as the dominant antibacterial constituent of Manuka (Leptospermum scoparium) honeys from New Zealand. Mol. Nutr. Food Res..

[40] Kato Y., Umeda N., Maeda A., Matsumoto D., Kitamoto N., Kikuzaki H. (2012). Identification of a Novel Glycoside, Leptosin, as a Chemical Marker of Manuka Honey. J. Agric. Food Chem..

[41] Jenkins R., Cooper R. (2012). Improving antibiotic activity against wound pathogens with manuka honey in vitro. PLoS ONE.

[42] Müller P., Alber D.G., Turnbull L., Schlothauer R.C., Carter D.A., Whitchurch C.B., Harry E.J. (2013). Synergism between Medihoney and Rifampicin against Methicillin-Resistant Staphylococcus aureus (MRSA). PLoS ONE.

[43] Gethin G., Cowman S., Conroy R. (2008). The impact of Manuka honey dressings on the surface pH of chronic wounds. Int. Wound J..

[44] Brudzynski K., Sjaarda C. (2014). Antibacterial compounds of Canadian honeys target bacterial cell wall inducing phenotype changes, growth inhibition and cell lysis that resemble action of β-lactam antibiotics. PLoS ONE.

[45] Mokaya H.O., Bargul J.L., Irungu J.W., Lattorff H.M.G. (2020). Bioactive constituents, in vitro radical scavenging and antibacterial activities of selected Apis mellifera honey from Kenya. Int. J. Food Sci. Technol..

[46] Cooper R.A., Molan P.C., Harding K.G. (2002). The sensitivity to honey of Gram-positive cocci of clinical significance isolated from wounds. J. Appl. Microbiol..

[47] Brudzynski K., Lannigan R. (2012). Mechanism of Honey Bacteriostatic Action Against MRSA and VRE Involves Hydroxyl Radicals Generated from Honey’s Hydrogen Peroxide. Front. Microbiol..

[48] Header E., Hashish A., ElSawy N., Alkushi A., El-Boshy M., Elsawy N. (2016). Gastroprotective effect of dietary honey against acetylsalicylate induced expermental ulcer in albino rat. Life Sci. J..

[49] Brown H., Metters G., Hitchings M., Wilkinson T., Sousa L., Cooper J., Dance H., Atterbury R., Jenkins R. (2020). Antibacterial and anti-virulence activity of manuka honey against genetically diverse Staphylococcus pseudintermedius. Appl. Environ. Microbiol..

[50] Alnaqdy A., Al-Jabri A., Al Mahrooqi Z., Nzeako B., Nsanze H. (2005). Inhibition effect of honey on the adherence of Salmonella to intestinal epithelial cells in vitro. Int. J. Food Microbiol..

[51] Badet C., Quero F. (2011). The in vitro effect of manuka honeys on growth and adherence of oral bacteria. Anaerobe.

[52] Alandejani T., Marsan J., Ferris W., Slinger R., Chan F. (2009). Effectiveness of honey on Staphylococcus aureus and Pseudomonas aeruginosa biofilms. Otolaryngol. Head Neck Surg..

[53] Cooper R.A., Wigley P., Burton N.F. (2000). Susceptibility of multiresistant strains of Burkholderia cepacia to honey. Lett. Appl. Microbiol..

[54] Wilkinson J.M., Cavanagh H.M.A. (2005). Antibacterial activity of 13 honeys against Escherichia coli and Pseudomonas aeruginosa. J. Med. Food..

[55] Moussa A., Noureddine D., Mohamed H.S., Abdelmelek M., Saad A. (2012). Antibacterial activity of various honey types of Algeria against Staphylococcus aureus and Streptococcus pyogenes. Asian Pac. J. Trop. Med..

[56] Lusby P.E., Coombes A.L., Wilkinson J.M. (2005). Bactericidal activity of different honeys against pathogenic bacteria. Arch Med. Res..

[57] Eick S., Schäfer G., Kwieciński J., Atrott J., Henle T., Pfister W. (2014). Honey a potential agent against Porphyromonas gingivalis: An in vitro study. BMC Oral Health.

[58] Bucekova M., Bugarova V., Godocikova J., Majtan J. (2020). Demanding New Honey Qualitative Standard Based on Antibacterial Activity. Foods.

[59] Combarros-Fuertes P.M., Estevinho L., Teixeira-Santos R.G., Rodrigues A., Pina-Vaz C., Fresno J.M., Tornadijo M.E. (2020). Antibacterial Action Mechanisms of Honey: Physiological Effects of Avocado, Chestnut, and Polyfloral Honey upon Staphylococcus aureus and Escherichia coli. Molecules.

[60] Adeleke O.E., Olaitan J.O., Okpekpe E.L. (2006). Comparative Antibacterial Activity of Honey and Gentamicin Against Escherichia Coli and Pseudomonas Aeruginosa. Ann. Burn. Fire Disasters.

[61] Al-Jabri A.A., Al-Hosni S.A., Nzeako B.C., Al-Mahrooqi Z.H., Nsanze H. (2005). Antibacterial activity of Omani honey alone and in combination with gentamicin. Saudi Med. J..

[62] Julianti E., Rajah K.K., Fidrianny I. (2017). Antibacterial Activity of Ethanolic Extract of Cinnamon Bark, Honey, and Their Combination Effects against Acne-Causing Bacteria. Sci. Pharm..

[63] Campeau M.E.M., Patel R. (2014). Antibiofilm Activity of Manuka Honey in Combination with Antibiotics. Int. J. Bacteriol..

[64] Liu M., Lu J., Müller P., Turnbull L., Burke C.M., Schlothauer R.C., Carter D.A., Whitchurch C.B., Harry E.J. (2014). Antibiotic-specific differences in the response of Staphylococcus aureus to treatment with antimicrobials combined with manuka honey. Front. Microbiol..

[65] Jenkins R., Cooper R. (2012). Synergy between oxacillin and manuka honey Sensitizes methicillin-resistant Staphylococcus aureus to oxacillin. J. Antimicrob. Chemother..

[66] Blaser G., Santos K., Bode U., Vetter H., Simon A. (2007). Effect of medical honey on wounds colonised or infected with MRSA. J. Wound Care.

[67] Boyanova L., Ilieva J., Gergova G., Vladimirov B., Nikolov R., Mitov I. (2015). Honey and green/black tea consumption may reduce the risk of Helicobacter pylori infection. Diagn. Microbiol. Infect. Dis..

[68] Yousaf I., Ishaq I., Hussain M.B., Inaam S., Saleem S., Qamar M.U. (2019). Antibacterial activity of Pakistani Beri honey compared with silver sulfadiazine on infected wounds: A clinical trial. J. Wound Care.

[69] Szweda P. (2017). Antimicrobial Activity of Honey [Internet]. Honey Analysis. IntechOpen. https://www.intechopen.com/books/honey-analysis/antimicrobial-activity-of-honey.

[70] Cooper R.A., Jenkins L., Henriques A.F.M., Duggan R.S., Burton N.F. (2010). Absence of bacterial resistance to medical-grade manuka honey. Eur. J. Clin. Microbiol. Infect. Dis..

[71] Combarros-Fuertes P., Fresno J.M., Estevinho M.M., Sousa-Pimenta M., Tornadijo M.E., Estevinho L.M. (2020). Honey: Another Alternative in the Fight against Antibiotic-Resistant Bacteria?. Antibiotics.

[72] Blair S.E., Cokcetin N.N., Harry E.J., Carter D.A. (2009). The unusual antibacterial activity of medical-grade Leptospermum honey: Antibacterial spectrum, resistance and transcriptome analysis. Eur. J. Clin. Microbiol. Infect. Dis..

[73] Hermanns R., Mateescu C., Thrasyvoulou A., Tananaki C., Wagener F., Cremers N. (2019). Defining the standards for medical grade honey. J. Apic. Res..

[74] Molan P.C., Allen K.L. (1996). The Effect of Gamma-irradiation on the Antibacterial Activity of Honey. J. Pharm. Pharmacol..

[75] Baracchi D., Francese S., Turillazzi S. (2011). Beyond the antipredatory defence: Honey bee venom function as a component of social immunity. Toxicon.

[76] Lee J.A., Son M.J., Choi J., Yun K.-J., Jun J.H., Lee M.S. (2014). Bee venom acupuncture for rheumatoid arthritis: A systematic review protocol. BMJ Open.

[77] Zhang S., Liu Y., Ye Y., Wang X.-R., Lin L.-T., Xiao L.-Y., Zhou P., Shi G.X., Liu C.Z. (2018). Bee venom therapy: Potential mechanisms and therapeutic applications. Toxicon.

[78] Wehbe R., Frangieh J., Rima M., El Obeid D., Sabatier J.-M., Fajloun Z. (2019). Bee Venom: Overview of Main Compounds and Bioactivities for Therapeutic Interests. Molecules.

[79] Wang L., Zhao X., Zhu C., Zhao Y., Liu S., Xia X., Liu X., Zhang H., Xu Y., Hang B. (2020). The antimicrobial peptide MPX kills Actinobacillus pleuropneumoniae and reduces its pathogenicity in mice. Vet Microbiol..

[80] Owen M.D., Pfaff L.A. (1995). Melittin synthesis in the venom system of the honey bee (Apis mellifera L.). Toxicon.

[81] Son D.J., Lee J.W., Lee Y.H., Song H.S., Lee C.K., Hong J.T. (2007). Therapeutic application of anti-arthritis, pain-releasing, and anti-cancer effects of bee venom and its constituent compounds. Pharm. Ther..

[82] Frangieh J., Salma Y., Haddad K., Mattei C., Legros C., Fajloun Z., El Obeid D. (2019). First Characterization of The Venom from Apis mellifera syriaca, A Honeybee from The Middle East Region. Toxins.

[83] Han S.M., Park K.K., Nicholls Y.M., Macfarlane N., Duncan G. (2013). Effects of honeybee (Apis mellifera) venom on keratinocyte migration in vitro. Pharm. Mag..

[84] Marques Pereira A.F., Albano M., Bérgamo Alves F.C., Murbach Teles Andrade B.F., Furlanetto A., Mores Rall V.L., Delazari D.S.L., de Oliveira O.R., Fernandes J.A. (2020). Influence of apitoxin and melittin from Apis mellifera bee on Staphylococcus aureus strains. Microb. Pathog..

[85] Ostroumova O.S., Efimova S.S., Malev V.V., Jeon K.W. (2015). Chapter Six Modifiers of Membrane Dipole Potentials as Tools for Investigating Ion Channel Formation and Functioning. International Review of Cell and Molecular Biology.

[86] Asthana N., Yadav S.P., Ghosh J.K. (2004). Dissection of antibacterial and toxic activity of melittin: A leucine zipper motif plays a crucial role in determining its hemolytic activity but not antibacterial activity. J. Biol. Chem..

[87] AL-Ani I., Zimmermann S., Reichling J., Wink M. (2015). Pharmacological synergism of bee venom and melittin with antibiotics and plant secondary metabolites against multi-drug resistant microbial pathogens. Phytomedicine.

[88] Yacoub T., Rima M., Karam M., Sabatier J.-M., Fajloun Z. (2020). Antimicrobials from Venomous Animals: An Overview. Molecules.

[89] Hegazi A., Abdel-Rahman E.H., Alfattah A. (2015). Antibacterial Activity of Bee Venom Collected from Apis Mellifera Carniolan Pure and Hybrid Races by Two Collection Methods. Undefined. /paper/Antibacterial-Activity-of-Bee-Venom-Collected-from-Hegazi-Abdel-Rahman/92bff1e2eff3d71ca4eb0ada4876e4b68d24d3fe.

[90] Socarras K., Theophilus P., Torres J., Gupta K., Sapi E. (2017). Antimicrobial Activity of Bee Venom and Melittin against Borrelia burgdorferi. Antibiotics.

[91] Jung B.-G., Lee J.-A., Park S.-B., Hyun P.-M., Park J.-K., Suh G.-H., Lee B.G. (2013). Immunoprophylactic Effects of Administering Honeybee (Apis melifera) Venom Spray against Salmonella Gallinarum in Broiler Chicks. J. Vet Med. Sci..

[92] Han S., Yeo J., Baek H., Lin S.-M., Meyer S., Molan P. (2009). Postantibiotic effect of purified melittin from honeybee (Apis mellifera) venom against Escherichia coli and Staphylococcus aureus. J. Asian Nat. Prod. Res..

[93] Picoli T., Peter C.M., Zani J.L., Waller S.B., Lopes M.G., Boesche K.N., Vargas G.D.Á., Hübner S.O., Fischer G. (2017). Melittin and its potential in the destruction and inhibition of the biofilm formation by Staphylococcus aureus, Escherichia coli and Pseudomonas aeruginosa isolated from bovine milk. Microb. Pathog..

[94] Wu X., Singh A.K., Wu X., Lyu Y., Bhunia A.K., Narsimhan G. (2016). Characterization of antimicrobial activity against Listeria and cytotoxicity of native melittin and its mutant variants. Colloids Surf. B Biointerfaces.

[95] Leandro L.F., Mendes C.A., Casemiro L.A., Vinholis A.H.C., Cunha W.R., de Almeida R., Martin C.H. (2015). Antimicrobial activity of apitoxin, melittin and phospholipase A₂ of honey bee (Apis mellifera) venom against oral pathogens. Acad. Bras. Cienc..

[96] Bitar L., Jundi D., Rima M., Al Alam J., Sabatier J.-M., Fajloun Z. (2019). Bee Venom PLA2 versus Snake Venom PLA2: Evaluation of Structural and Functional Properties. Venoms Toxins.

[97] Choi J.H., Jang A.Y., Lin S., Lim S., Kim D., Park K., Han S.M., Yeo J.H., Seo H.S. (2015). Melittin, a honeybee venom-derived antimicrobial peptide, may target methicillin-resistant Staphylococcus aureus. Mol. Med. Rep..

[98] Jamasbi E., Batinovic S., Sharples R.A., Sani M.-A., Robins-Browne R.M., Wade J.D., Separovic F., Hossain M.A. (2014). Melittin peptides exhibit different activity on different cells and model membranes. Amino Acids.

[99] Park D., Jung J.W., Lee M.O., Lee S.Y., Kim B., Jin H.J., Kim J., Ahn Y.J., Lee K.W., Song Y.S. (2014). Functional characterization of naturally occurring melittin peptide isoforms in two honey bee species, Apis mellifera and Apis cerana. Peptides.

[100] Akbari R., Hakemi vala M., Hashemi A., Aghazadeh H., Sabatier J.-M., Pooshang Bagheri K. (2018). Action mechanism of melittin-derived antimicrobial peptides, MDP1 and MDP2, de novo designed against multidrug resistant bacteria. Amino Acids.

[101] Fadl A. (2018). Antibacterial and antibiofilm effects of bee venom from (Apis mellifera) on multidrug-resistant bacteria (MDRB). Al-Azhar J. Pharm. Sci..

[102] Han S.M., Kim J.M., Hong I.P., Woo S.O., Kim S.G., Jang H.R., Pak S.C. (2016). Antibacterial Activity and Antibiotic-Enhancing Effects of Honeybee Venom against Methicillin-Resistant Staphylococcus aureus. Molecules.

[103] Al-Safar M., Salman J. (2018). Antibacterial activity of bee venom against multidrug resistant Acinetobacter baumannii locally isolates. Int. J. Res. Pharm. Sci..

[104] Akbari R., Hakemi-Vala M., Pashaie F., Bevalian P., Hashemi A., Pooshang Bagheri K. (2019). Highly Synergistic Effects of Melittin with Conventional Antibiotics Against Multidrug-Resistant Isolates of Acinetobacter baumannii and Pseudomonas aeruginosa. Microb. Drug Resist..

[105] Giacometti A., Cirioni O., Kamysz W., D’Amato G., Silvestri C., Del Prete M.S., Łukasiak J., Scalise G. (2003). Comparative activities of cecropin A, melittin, and cecropin A-melittin peptide CA(1-7)M(2-9)NH2 against multidrug-resistant nosocomial isolates of Acinetobacter baumannii. Peptides.

[106] Pucca M.B., Cerni F.A., Oliveira I.S., Jenkins T.P., Argemí L., Sørensen C.V., Ahmadi S., Barbosa J.E., Laustsen A.H. (2019). Bee Updated: Current Knowledge on Bee Venom and Bee Envenoming Therapy. Front. Immunol..

[107] Cherniack E.P., Govorushko S. (2018). To bee or not to bee: The potential efficacy and safety of bee venom acupuncture in humans. Toxicon.

[108] Almeida R.A.M., Olivo T.E.T., Mendes R.P., Barraviera S.R.C.S., Souza L., Martins J.G., Hashimoto M., Fabris V.E., Ferreira Junior R.S., Barraviera B. (2011). Africanized honeybee stings: How to treat them. Rev. Soc. Bras. Med. Trop..

[109] Kragballe K. (1989). Topical corticosteroids: Mechanisms of action. Acta Derm. Venereol. Suppl. Stockh..

[110] Fitzgerald K.T., Flood A.A. (2006). Hymenoptera stings. Clin. Tech. Small Anim. Pr..

[111] Kim H., Park S.-Y., Lee G. (2019). Potential Therapeutic Applications of Bee Venom on Skin Disease and Its Mechanisms: A Literature Review. Toxins.

[112] Han S.M., Lee K.G., Pak S.C. (2013). Effects of cosmetics containing purified honeybee (Apis mellifera L.) venom on acne vulgaris. J. Integr. Med..

[113] Park J.H., Yim B.K., Lee J.-H., Lee S., Kim T.-H. (2015). Risk Associated with Bee Venom Therapy: A Systematic Review and Meta-Analysis. PLoS ONE.

[114] Popova M., Silici S., Kaftanoglu O., Bankova V. (2005). Antibacterial activity of Turkish propolis and its qualitative and quantitative chemical composition. Phytomedicine.

[115] Crane E. (2015). A short history of knowledge about honey bees (Apis) up to 1800. Bee World..

[116] Galeotti F., Maccari F., Fachini A., Volpi N. (2018). Chemical Composition and Antioxidant Activity of Propolis Prepared in Different Forms and in Different Solvents Useful for Finished Products. Foods.

[117] Pimenta H.C., Violante I.M.P., Musis C.R., de Borges Á.H., Aranha A.M.F. (2015). In vitro effectiveness of Brazilian brown propolis against Enterococcus faecalis. Braz. Oral Res..

[118] Miguel M.G., Nunes S., Dandlen S.A., Cavaco A.M., Antunes M.D. (2010). Phenols and antioxidant activity of hydro-alcoholic extracts of propolis from Algarve, South of Portugal. Food Chem. Toxicol..

[119] Inui S., Hatano A., Yoshino M., Hosoya T., Shimamura Y., Masuda S., Ahn M.R., Tazawa S., Araki Y., Kumazawa S. (2014). Identification of the phenolic compounds contributing to antibacterial activity in ethanol extracts of Brazilian red propolis. Nat. Prod. Res..

[120] Bastos E.M.A., Simone M., Jorge D.M., Soares A.E.E., Spivak M. (2008). In vitro study of the antimicrobial activity of Brazilian propolis against Paenibacillus larvae. J. Invertebr. Pathol..

[121] Veiga R.S., Mendonça S.D., Mendes P.B., Paulino N., Mimica M.J., Netto A.A.L., Lira I.S., López B.G., Negrão V., Marcucci M.C. (2017). Artepillin C and phenolic compounds responsible for antimicrobial and antioxidant activity of green propolis and Baccharis dracunculifolia DC. J. Appl. Microbiol..

[122] Yoshimasu Y., Ikeda T., Sakai N., Yagi A., Hirayama S., Morinaga Y., Furukawa S., Nakao R. (2018). Rapid Bactericidal Action of Propolis against Porphyromonas gingivalis. J. Dent. Res..

[123] Seibert J.B., Bautista-Silva J.P., Amparo T.R., Petit A., Pervier P., dos Santos Almeida J.C., Azevedo M.C., Silveira B.M., Brandão G.C., de Souza G.H.B. (2019). Development of propolis nanoemulsion with antioxidant and antimicrobial activity for use as a potential natural preservative. Food Chem..

[124] Veloz J.J., Alvear M., Salazar L.A. (2019). Antimicrobial and Antibiofilm Activity against Streptococcus mutans of Individual and Mixtures of the Main Polyphenolic Compounds Found in Chilean Propolis. Biomed Res. Int..

[125] Kharsany K., Viljoen A., Leonard C., van Vuuren S. (2019). The new buzz: Investigating the antimicrobial interactions between bioactive compounds found in South African propolis. J. Ethnopharmacol..

[126] Okińczyc P., Paluch E., Franiczek R., Widelski J., Wojtanowski K.K., Mroczek T., Krzyżanowska B., Skalicka-Woźniak K., Sroka Z. (2020). Antimicrobial activity of Apis mellifera L. and Trigona sp. propolis from Nepal and its phytochemical analysis. Biomed. Pharmacother..

[127] Wojtyczka R.D., Dziedzic A., Idzik D., Kępa M., Kubina R., Kabała-Dzik A., Smoleń-Dzirba J., Stojko J., Sajewicz M., Wąsik T.J. (2013). Susceptibility of Staphylococcus aureus Clinical Isolates to Propolis Extract Alone or in Combination with Antimicrobial Drugs. Molecules.

[128] Seidel V., Peyfoon E., Watson D.G., Fearnley J. (2008). Comparative study of the antibacterial activity of propolis from different geographical and climatic zones. Phytother Res..

[129] Kilic A., Baysallar M., Besirbellioglu B., Salih B., Sorkun K., Tanyuksel M. (2005). In Vitro Antimicrobial Activity of Propolis against Methicillin-Resistant Staphylococcus Aureus and Vancomycin-Resistant Enterococcus Faecium. https://avesis.hacettepe.edu.tr/yayin/3ef4193c-cc98-4711-bfca-d5a21f1fa7ee/in-vitro-antimicrobial-activity-of-propolis-against-methicillin-resistant-staphylococcus-aureus-and-vancomycin-resistant-enterococcus-faecium.

[130] Koru O., Toksoy F., Acikel C.H., Tunca Y.M., Baysallar M., Uskudar Guclu A., Akca E., Ozkok Tuylu A., Sorkun K., Tanyuksel M. (2007). In vitro antimicrobial activity of propolis samples from different geographical origins against certain oral pathogens. Anaerobe.

[131] Shabbir A., Rashid M., Tipu H.N. (2016). Propolis, A Hope for the Future in Treating Resistant Periodontal Pathogens. Cureus.

[132] Boyanova L., Kolarov R., Gergova G., Mitov I. (2006). In vitro activity of Bulgarian propolis against 94 clinical isolates of anaerobic bacteria. Anaerobe.

[133] Fernandes Júnior A., Balestrin E.C., Betoni J.E.C., Orsi R., de Cunha M., Montelli A.C. (2005). Propolis: Anti-Staphylococcus aureus activity and synergism with antimicrobial drugs. Mem. Inst. Oswaldo Cruz..

[134] Orsi R.O., Fernandes A., Bankova V., Sforcin J.M. (2012). The effects of Brazilian and Bulgarian propolis in vitro against Salmonella Typhi and their synergism with antibiotics acting on the ribosome. Nat. Prod. Res..

[135] AL-Ani I., Zimmermann S., Reichling J., Wink M. (2018). Antimicrobial Activities of European Propolis Collected from Various Geographic Origins Alone and in Combination with Antibiotics. Medicines.

[136] Ali B.M.M., Ghoname N.F., Hodeib A., Elbadawy M. (2015). Significance of topical propolis in the treatment of facial acne vulgaris. Egypt. J. Dermatol. Venerol..

[137] Darwita R.R., Finisha A., Nur Wahyuni H., Ghina S., Muhammad R., Satyanegara A., Setiawati F., Adiatman M. (2018). The effectiveness of propolis fluoride application in inhibiting dental caries activity in school children age 6-9 years old. Int. J. Appl. Pharm..

[138] Gebara E.C.E., Pustiglioni A.N. (2003). Propolis Extract as an Adjuvant to Periodontal Treatment. Oral Health.

[139] Fatrcová-Šramková K., Nôžková J., Kačániová M., Máriássyová M., Rovná K., Stričík M. (2013). Antioxidant and antimicrobial properties of monofloral bee pollen. J. Environ. Sci. Health B.

[140] Bakour M., Fernandes Â., Barros L., Sokovic M., Ferreira I.C.F.R., Badiaa L. (2019). Bee bread as a functional product: Chemical composition and bioactive properties. LWT.

[141] Kaškonienė V., Adaškevičiūtė V., Kaškonas P., Mickienė R., Maruška A. (2020). Antimicrobial and antioxidant activities of natural and fermented bee pollen. Food Biosci..

[142] Schmickl T., Crailsheim K. (2001). Cannibalism and early capping: Strategy of honeybee colonies in times of experimental pollen shortages. J. Comp. Physiol. A.

[143] Taha E.-K.A., Al-Kahtani S., Taha R. (2019). Protein content and amino acids composition of bee-pollens from major floral sources in Al-Ahsa, eastern Saudi Arabia. Saudi J. Biol. Sci..

[144] Silva G.R., Natividade T.B., Camara C.A., Silva E.M.S., Santos F., Silva T.M.S. (2014). Identification of Sugar, Amino Acids and Minerals from the Pollen of Jandaíra Stingless Bees (*Melipona subnitida*). Food Nutr. Sci..

[145] Abouda Z., Zerdani I., Kalalou I., Faid M., Ahami M.T. (2011). The Antibacterial Activity of Moroccan Bee Bread and Bee-Pollen (Fresh and Dried) against Pathogenic Bacteria. Res. J. Microbiol..

[146] Kroyer G., Hegedus N. (2001). Evaluation of bioactive properties of pollen extracts as functional dietary food supplement. Innov. Food Sci. Emerg. Technol..

[147] Morais M., Moreira L., Feás X., Estevinho L.M. (2011). Honeybee-collected pollen from five Portuguese Natural Parks: Palynological origin, phenolic content, antioxidant properties and antimicrobial activity. Food Chem. Toxicol..

[148] Erkmen O., Ozcan M.M. (2008). Antimicrobial effects of Turkish propolis, pollen, and laurel on spoilage and pathogenic food-related microorganisms. J. Med. Food.

[149] Kacaniova M. (2015). Antimicrobial Activity of Bee Collected Pollen against Clostridia. Sci. Pap. Anim. Sci. Biotechnol..

[150] Cabrera C., Montenegro G. (2013). Pathogen control using a natural Chilean bee pollen extract of known botanical origin. Cienc. Investig. Agrar..

[151] Karadal F., Onmaz N.E., Abay S., Yildirim Y., Al S., Tatyuz I., Ackay A. (2018). A Study of Antibacterial and Antioxidant Activities of Bee Products: Propolis, Pollen and Honey Samples. Ethiop. J. Health Dev..

[152] Šimunović K., Abramovi H., Lilek N., Angelova M., Podržaj L., Smole Možina S. (2019). Microbiological quality, antioxidantive and antimicrobial properties of Slovenian bee pollen. AGROFOR.

[153] Kacaniova M., Vuković N., Chlebo R., Haščík P., Rovná K., Cubon J., Dżugan M., PASTERNAKIEWICZ A. (2012). The antimicrobial activity of honey, bee pollen loads and beeswax from Slovakia. Arch. Biol. Sci..

[154] Campos M., Bogdanov S., Almeida-Muradian L., Szczesna T., Mancebo Y., Frigerio C., Ferreira F. (2008). Pollen composition and standardisation of analytical methods. J. Apic. Res. Bee World.

[155] Freire K.R.L., Lins A.C.S., Dórea M.C., Santos F.A.R., Camara C.A., Silva T.M.S. (2012). Palynological Origin, Phenolic Content, and Antioxidant Properties of Honeybee-Collected Pollen from Bahia, Brazil. Molecules.

[156] Velásquez P., Rodríguez K., Retamal M., Giordano A., Valenzuela L., Montenegro G. (2017). Relation between composition, antioxidant and antibacterial activities and botanical origin of multi-floral bee pollen. J. Appl. Bot. Food Qual..

[157] Khider M., Elbanna K., Mahmoud A., Owayss A. (2013). Egyptian Honeybee Pollen as Antimicrobial, Antioxidant Agents, and Dietary Food Supplements. Food Sci. Biotechnol..

[158] Buttstedt A., Moritz R.F., Erler S. (2013). More than royal food Major royal jelly protein genes in sexuals and workers of the honeybee Apis mellifera. Front. Zool..

[159] Fujita T., Kozuka-Hata H., Ao-Kondo H., Kunieda T., Oyama M., Kubo T. (2013). Proteomic analysis of the royal jelly and characterization of the functions of its derivation glands in the honeybee. J. Proteome Res..

[160] Nagai T., Inoue R. (2004). Preparation and functional properties of water extract and alkaline extract of royal jelly. Food Chem..

[161] Bíliková K., Hanes J., Nordhoff E., Saenger W., Klaudiny J., Simúth J. (2002). Apisimin, a new serine-valine-rich peptide from honeybee (Apis mellifera L.) royal jelly: Purification and molecular characterization. FEBS Lett..

[162] Buttstedt A., Moritz R.F.A., Erler S. (2014). Origin and function of the major royal jelly proteins of the honeybee (Apis mellifera) as members of the yellow gene family. Biol. Rev. Camb. Philos. Soc..

[163] Furusawa T., Rakwal R., Nam H.W., Shibato J., Agrawal G.K., Kim Y.S., Ogawa Y., Yoshida Y., Kouzuma Y., Masuo Y. (2008). Comprehensive royal jelly (RJ) proteomics using one- and two-dimensional proteomics platforms reveals novel RJ proteins and potential phospho/glycoproteins. J. Proteome Res..

[164] Scarselli R., Donadio E., Giuffrida M.G., Fortunato D., Conti A., Balestreri E., Felicioli R., Pinzauti M., Sabatini A.G., Felicioli A. (2005). Towards royal jelly proteome. Proteomics.

[165] Hanes J., Šimuth J. (1992). Identification and partial characterization of the major royal jelly protein of the honey bee (Apis mellifera L.). J. Apic. Res..

[166] Kamakura M. (2011). Royalactin induces queen differentiation in honeybees. Nature.

[167] Šimúth J. (2001). Some properties of the main protein of honeybee (Apis mellifera) royal jelly. Apidologie.

[168] Fontana R., Mendes M.A., de Souza B.M., Konno K., César L.M.M., Malaspina O., Palma M.S. (2004). Jelleines: A family of antimicrobial peptides from the Royal Jelly of honeybees (Apis mellifera). Peptides.

[169] Bucekova M., Majtan J. (2016). The MRJP1 honey glycoprotein does not contribute to the overall antibacterial activity of natural honey. Eur. Food Res. Technol..

[170] Bíliková K., Mirgorodskaya E., Bukovská G., Gobom J., Lehrach H., Simúth J. (2009). Towards functional proteomics of minority component of honeybee royal jelly: The effect of post-translational modifications on the antimicrobial activity of apalbumin2. Proteomics.

[171] Okamoto I., Taniguchi Y., Kunikata T., Kohno K., Iwaki K., Ikeda M., Kurimoto M. (2003). Major royal jelly protein 3 modulates immune responses in vitro and in vivo. Life Sci..

[172] Rosmilah M., Shahnaz M., Patel G., Lock J., Rahman D., Masita A., Noormalin A. (2008). Characterization of major allergens of royal jelly Apis mellifera. Trop Biomed..

[173] Fujiwara S., Imai J., Fujiwara M., Yaeshima T., Kawashima T., Kobayashi K. (1990). A potent antibacterial protein in royal jelly. Purification and determination of the primary structure of royalisin. J. Biol. Chem..

[174] Bilikova K., Wu G., Šimúth J. (2001). Isolation of a peptide fraction from honeybee royal jelly as a potential antifoulbrood factor. Apidologie.

[175] Bachanov K., Klaudiny J., Kopernick J., Šimúth J. (2002). Identification of honeybee peptide active against Paenibacillus larvae larvae through bacterial growth-inhibition assay on polyacrylamide gel. Apidologie.

[176] Klaudiny J., Bachanová K., Kohútová L., Dzúrová M., Kopernický J., Majtan J. (2012). Expression of larval jelly antimicrobial peptide defensin1 in Apis mellifera colonies. Biologia.

[177] Shen L., Liu D., Li M., Jin F., Din M., Parnell L.D., Lai C.Q. (2012). Mechanism of action of recombinant acc-royalisin from royal jelly of Asian honeybee against Gram-positive bacteria. PLoS ONE.

[178] Zhou J., Xue X., Li Y., Zhang J., Zhao J. (2007). Optimized Determination Method for trans-10-Hydroxy-2-Decenoic Acid Content in Royal Jelly by High-Performance Liquid Chromatography with an Internal Standard. J. Aoac Int..

[179] Šedivá M., Laho M., Kohútová L., Mojžišová A., Majtán J., Klaudiny J. (2018). 10-HDA, A Major Fatty Acid of Royal Jelly, Exhibits pH Dependent Growth-Inhibitory Activity Against Different Strains of Paenibacillus larvae. Molecules.

[180] Bc B., Cs H., Ct H., Yo B. (1995). Liquid chromatographic determination of trans-10-hydroxy-2-decenoic acid content of commercial products containing royal jelly. J. Aoac Int..

[181] Blum M.S., Novak A.F., Taber S. (1959). 10-Hydroxy-delta 2-decenoic acid, an antibiotic found in royal jelly. Science.

[182] Moselhy W.A., Fawzy A.M., Kamel A.A. (2013). An evaluation of the potent antimicrobial effects and unsaponifiable matter analysis of the royal jelly. Life Sci. J..

[183] Yousefi B., Ghaderi S., Rezapoor-Lactooyi A., Amiri N., Verdi J., Shoae-Hassani A. (2012). Hydroxy decenoic acid down regulates gtfB and gtfC expression and prevents Streptococcus mutans adherence to the cell surfaces. Ann. Clin. Microbiol. Antimicrob..

[184] Melliou E., Chinou I. (2005). Chemistry and bioactivity of royal jelly from Greece. J. Agric. Food Chem..

[185] Celeste García M., Silvia Finola M., Miguel Marioli J. (2021). Antibacterial activity of Royal Jelly against bacteria capable of infecting cutaneous wounds. Int. Bee Res. Assoc..

[186] Ratanavalachai T., Wongchai V. (2002). Antibacterial Activitv of Intact Roval Jelly, Its Lipid Extract and Its Defatted Extract. Sci. Technol. Asia.

[187] Khosla A., Gupta S.J., Jain A., Shetty D.C., Sharma N. (2020). Evaluation and comparison of the antimicrobial activity of royal jelly A holistic healer against periodontopathic bacteria: An in vitro study. J. Indian Soc. Periodontol..

[188] Boukraâ L., Meslem A., Mokhtar B., Hammoudi S. (2009). Synergistic Effect of Starch and Royal Jelly Against Staphylococcus aureus and Escherichia coli. J. Altern. Complement. Med..

[189] Boukraa L. (2008). Additive activity of royal jelly and honey against Pseudomonas aeruginosa. Altern. Med. Rev..

[190] Romanelli A., Moggio L., Montella R.C., Campiglia P., Iannaccone M., Capuano F. (2011). Peptides from Royal Jelly: Studies on the antimicrobial activity of jelleins, jelleins analogs and synergy with temporins. J. Pept. Sci..

[191] Al-Masaudi S.B., Hussain M.B., Al-Maaqar S.M., Al Jaouni S., Harakeh S. (2020). In vitro antibacterial activity of honey against multidrug-resistant Shigella sonnei. Complement.Ther. Clin. Pract..

[192] Ghramh H.A., Khan K.A., Alshehri A.M.A. (2019). Antibacterial potential of some Saudi honeys from Asir region against selected pathogenic bacteria. Saudi J. Biol. Sci..

[193] Park S.H., Kim Y.K., Kim M.S., Lee S.H. (2020). Antioxidant and Antibacterial Properties of Hovenia (Hovenia dulcis) Monofloral Honey Produced in South Korea. Food Sci. Anim. Resour..

[194] Al-Jabri A.A., Nzeako B., Al Mahrooqi Z., Al Naqdy A., Nsanze H. (2003). In vitro antibacterial activity of Omani and African honey. Br. J. Biomed. Sci..

[195] Sherlock O., Dolan A., Athman R., Power A., Gethin G., Cowman S. (2010). Comparison of the antimicrobial activity of Ulmo honey from Chile and Manuka honey against methicillin-resistant Staphylococcus aureus, Escherichia coli and Pseudomonas aeruginosa. BMC Complement. Altern Med..

[196] Biological Activities and Chemical Composition of Three Honeys of Different Types from Anatolia ScienceDirect. https://www.sciencedirect.com/science/article/abs/pii/S0308814605008964.

[197] Miorin P.L., Junior N.C.L., Custodio A.R., Bretz W.A., Marcucci M.C. (2003). Antibacterial activity of honey and propolis from Apis mellifera and Tetragonisca angustula against Staphylococcus aureus. J. Appl. Microbiol..

[198] French V.M., Cooper R.A., Molan P.C. (2005). The antibacterial activity of honey against coagulase-negative staphylococci. J. Antimicrob. Chemother..

[199] Lin S.M., Molan P.C., Cursons R.T. (2009). The in vitro susceptibility of Campylobacter spp. to the antibacterial effect of manuka honey. Eur. J. Clin. Microbiol. Infect. Dis..

[200] Al-Kafaween M.A., Al-Jamal H.A.N., Hilmi A.B.M., Elsahoryi N.A., Jaffar N., Zahri M.K. (2020). Antibacterial properties of selected Malaysian Tualang honey against Pseudomonas aeruginosa and Streptococcus pyogenes. Iran J. Microbiol..

[201] Cooper R.A., Halas E., Molan P.C. (2002). The efficacy of honey in inhibiting strains of Pseudomonas aeruginosa from infected burns. J. Burn. Care Rehabil..

[202] Rabie E., Serem J.C., Oberholzer H.M., Gaspar A.R.M., Bester M.J. (2016). How methylglyoxal kills bacteria: An ultrastructural study. Ultrastruct. Pathol..

